# Who Leads? Who Follows? Measuring Issue Attention and Agenda Setting by Legislators and the Mass Public Using Social Media Data

**DOI:** 10.1017/S0003055419000352

**Published:** 2019-07-12

**Authors:** Pablo BarberÁ, Andreu Casas, Jonathan Nagler, Patrick J. Egan, Richard Bonneau, John T. Jost, Joshua A. Tucker

**Affiliations:** 1 University of Southern California; 2 New York University

## Abstract

Are legislators responsive to the priorities of the public? Research demonstrates a strong correspondence between the issues about which the public cares and the issues addressed by politicians, but conclusive evidence about who leads whom in setting the political agenda has yet to be uncovered. We answer this question with fine-grained temporal analyses of Twitter messages by legislators and the public during the 113th US Congress. After employing an unsupervised method that classifies tweets sent by legislators and citizens into topics, we use vector autoregression models to explore whose priorities more strongly predict the relationship between citizens and politicians. We find that legislators are more likely to follow, than to lead, discussion of public issues, results that hold even after controlling for the agenda-setting effects of the media. We also find, however, that legislators are more likely to be responsive to their supporters than to the general public.

## INTRODUCTION

An enduring topic in the study of democratic polities is how responsive governments are to the preferences of the public. Two main lines of inquiry lead this research: Do politicians respond to the issue priorities of the public (Edwards and Wood 1999; Jones and Baumgartner 2004; Neundorf and Adams 2018; Sulkin 2005)? And, if so, do they reflect the policy preferences that citizens have on these issues (Caughey and Warshaw 2018; Page and Shapiro 1983; Soroka and Wlezien 2009; Stimson, Mackuen, and Erikson 1995)? Our manuscript focuses on the first of these two questions, because while a correspondence between public and political agendas has definitively been shown to exist, there is still high uncertainty about who leads and who follows in the agenda-setting process. Evidence is even more scant on the important question of which citizens have the strongest ability to set political agendas: the general public (Downs 1957), attentive citizens (Aldrich 1995; Arnold 1990), or politicians’ own supporters (Egan 2013; Kastellec et al. 2015).

We aim to shed new light on these classic and relevant political science questions by analyzing the issues to which members of the US Congress and the American public pay attention. Although determining whether politicians also follow constituents’ issue preferences and priorities on the policies they *implement*—and, if so, *which* constituents’ issue preferences—is of equal relevance to say the least, “policy actions cannot be taken unless attention is directed at the matter” (Jones and Baumgartner 2004, 2). Hence, disentangling whether politicians devote more time discussing an issue after attention to that issue by the public increases is a first and crucial step toward fully understanding political representation in the United States.^1^

We believe the lack of clear findings on who leads public opinion is partly a function of data limitations, as time and issue units available for previous studies did not allow for sufficiently granular measurement of the relationship between politicians’ and the public’s agendas. Most existing research relies on monthly survey data (typically Gallup’s “Most Important Problem” [MIP] question) to measure the public agenda. However, in our 24-hour media environment, politicians and the public are constantly adjusting the issues to which they devote attention, which means that changes in attention allocation are likely to happen within monthly survey waves. Hence, while survey data allow us to observe whether the public and political agendas covary, they provide limited information on which one moves first. Moreover, existing analyses trace attention to issue categories that are very broad (such as “the economy” or “immigration”), which has the advantage of facilitating comparisons across long periods of time and units, such as states and countries, but can make it difficult to uncover who leads public opinion by grouping together issues that are in fact quite different.^2^

In an effort to address previous data limitations, we pursue a novel empirical strategy by using the microblogging social media platform Twitter to measure the expressed agenda of legislators and the American public. To be clear, our goal is not to assess whether a social media platform such as Twitter is a useful agenda setting tool for politicians or the public but rather to use their “tweets” as a proxy to measure attention being paid to political issues. We are reassured in pursuing such a measurement strategy because virtually all members of the US Congress are active Twitter users and their tweets have been shown to constitute a standardized representation of their expressed issue agenda (Casas and Morar 2015). Moreover, the issues Americans discuss on social media are highly correlated with other measures of issue salience such as the MIP survey question (O’Connor et al. 2010).^3^

Twitter data provide two main advantages to address the questions at hand. First, the data allow us to measure public and political agendas using the same source: both members of Congress and their constituents are present on the platform, sending tweets that have the same format and symbolic references such as hashtags. Second, the high granularity of the data allows us to observe swiftly changing temporal patterns in topic salience. We are therefore able to pinpoint with precision the extent to which politicians allocate attention to different issues before or after shifts in issue attention by the public (or whether they devote attention to the issue at all). Although previous work has used Twitter data to evaluate the issues to which politicians and constituents pay attention (particularly, the work by Lilly Hemphill and colleagues: e.g., Hemphill and Roback (2014) and Shapiro and Hemphill (2017)), this work has examined a limited number of issues and has primarily focused on issue *congruence* rather than issue *responsiveness*.

We first analyze all tweets sent by members of the 113th Congress from January 2013 to December 2014. Using a Latent Dirichlet Allocation (LDA) model, we extract 100 topics that represent the diversity of issues legislators discuss on the social networking site. We show that this method is able to classify legislators’ tweets into a set of validated topics that exhibit meaningful variation over time and across parties. We then employ a vector autoregression (VAR) approach to explore the extent to which legislators’ expressed political agendas adapt after a change in issue attention by three different subgroups of the public: partisans, people who are particularly attentive to politics, and a random sample of US Twitter users. Our tests examine the extent to which changes in issue attention by these groups of citizens predict policy makers’ agendas.

Our observational analysis is necessarily of a descriptive rather than a causal nature. Our VAR approach takes advantage of multiple lags of data to distinguish when groups lead conversations about particular topics and by contrast when they are joining debates that are already established, but it cannot rule out confounding by unobservable factors. Nevertheless, our analyses provide revealing information about the configuration of political agendas and public opinion in the United States. Further, it allows us to ascertain in ways not previously possible whether observable data conform to empirical implications of theories specifying how the agendas of different sets of actors impact those of others, providing corroborative evidence for some of these theories.

Our findings show definitively that members of Congress are more likely to follow the issue priorities of the public than to lead them. However, this responsiveness is limited in ways that reinforce polarization and inequality. Lawmakers are more likely to change their behavior after shifts in attention by party supporters, as previous work leads us to expect (Bawn et al. 2012; Clinton 2006; Egan 2013; Kastellec et al. 2015; Shapiro et al. 1990). To a lesser extent, politicians are also responsive to the issue priorities of attentive citizens over those less inclined to follow politics (Aldrich 1995; Arnold 1990). But despite well-established models predicting that politicians should reflect the priorities of the general public (Downs 1957), we find little evidence for this. Our findings also suggest that mainstream media is in part to blame for this inequality on issue responsiveness: mass media are more likely to cover those issues that are of interest to partisans, and they often lead the political agenda.

## POLITICIANS’ RESPONSIVENESS TO THE PUBLIC’S PRIORITIES

Empirical studies on policy (not issue) responsiveness have substantially advanced due to great innovations in data collection and measurement (Burstein 2014; Caughey and Warshaw 2018; Gilens 2012; Lax and Phillips 2011; Soroka and Wlezien 2009; Tausanovitch and Warshaw 2014), but without a more clear understanding of issue responsiveness, an evaluation of the extent to which governments are responsive to their citizens is incomplete. As Jones and Baumgartner (2004) note, “How representative is a legislative action that matches the policy preferences of the public on a low priority issue but ignores high priority issues?” (p. 2). For politicians to be truly responsive to the public, they first need to pay attention to the issues constituents deem relevant, and then their actions must reflect people’s preferences on those issues.

Research on agenda setting and political responsiveness in the United States has found a strong relationship between the issue priorities of the public and the agenda of members of Congress (Baumgartner and Jones 1993). For issues such as the economy, health, environment, and foreign trade, changes in public issue salience (measured using Gallup’s long-standing MIP question) correlate at high levels with changes in political attention (measured as the proportion of Congressional hearings on the same issue) (Jones and Baumgartner 2004).

However, existing studies on issue responsiveness do not clearly address a very important question: who leads whom (Page 1994)? Are policy makers more likely to follow than to lead changes in issue attention by their constituents, or is it the other way around? Research indicates that both scenarios are possible, but it is unclear who (if any) has the largest capacity to lead the issue agenda of the other.

On the one hand, research on policy responsiveness argues that politicians have strong incentives to be responsive to the preferences of the public (Erikson, Mackuen, and Stimson 2002; Geer 1996; Stimson, Mackuen, and Erikson 1995). Building on the “retrospective voting” idea, (Campbell, Dettrey, and Yin 2010), scholars, such as Stimson, Mackuen, and Erikson (1995), argue (and find) that electorally oriented politicians update their preferences to maximize reelection prospects once they perceive a shift in public opinion: “when politicians perceive public opinion to change, they adapt their behavior to please their constituents” (p. 545).Canes-Wrone and Shotts (2004) also show that public opinion can influence the preferences of political figures, such as the president, particularly on issues directly related to people’s daily life. Overall, this literature suggests that politicians are responsive to public priorities and leads to the expectation that **(H1) the public’s priorities predict the issues to which members of Congress subsequently pay attention.**

On the other hand, another body of research argues the opposite. Building on the image of “policy-oriented” politicians, scholars argue that most politicians are mainly motivated by policy goals rather than by the goal of seeking reelection (Jacobs and Shapiro 2000). Research shows that most citizens are not interested in (Hibbing and Theiss-Morse 2002) and know very little (Delli Carpini and Keeter 1996) about politics, and that instead of evaluating politicians based on their past actions and performance, they make decisions based on group attachments (Campbell et al. 1960) and elite cues (Lupia, McCubbins, and Arthur 1998; Sniderman, Brody, and Tetlock 1993). Authors such as Lawrence R. Jacobs, Robert Y. Shapiro, and Benjamin I. Page draw on this literature and their own empirical evidence to show that policy-oriented politicians take advantage of people’s political disconnect to set the agenda to their liking. In interviews with administration officials, the authors are repeatedly told that the government tracks public opinion “not to ‘pander’ but to educate, lead, or otherwise influence public attitudes” (Jacobs and Shapiro 1997, 3). Overall, this other body of research leads to the expectation that **(H2) members of Congress initiate debates about issues that are subsequently followed by the public.**

Thus, there are good reasons to believe that politicians follow the issue preferences of the public, but also that the public responds to politicians’ issue priorities. But who has the strongest ability to lead the issue agenda of the other? A primary contribution of our analysis will be to evaluate the magnitude of these effects to determine who has the largest agenda-setting effect (if any). We explore this question without a theoretical preference for either hypothesis, but rather as an open debate that must be addressed to truly evaluate the nature of political responsiveness in the American democratic system (Burstein 2003; Page 1994).

## MODELS OF RESPONSIVENESS

Beyond whether politicians or the public have the largest agenda-setting effect, a second question is also crucial for advancing a more complete picture of issue responsiveness in the United States: to whom should we expect members of Congress to be responsive?

Despite a substantial number of studies on the issue, the answer is not as straightforward as one might think. As Burstein (2003, 30) points out, “one might hope that 20 years of research would enhance the credibility of some [political responsiveness] theories and reduce that of others. But this does not seem to have happened.” In particular, we observe three main theoretical models to pose three different answers to our question of interest. We call them here the *Downsian*, the *Attentive*, and the *Supporter* models.^4^

In *An Economic Theory of Democracy*, Downs (1957) argued that, in a bipartisan democratic system, policy makers interested in reelection should be responsive to the median voter or “centrist opinion” (Jacobs and Shapiro 2000). The implications for political responsiveness are easier to envision from a policy—as opposed to from an issue responsiveness— perspective: members of Congress should adopt the policy preference of their median constituent. Following the same logic, we should also expect legislators to increase their chances of reelection by focusing on issues that a majority of the general public deems relevant. Some strongly disagree and argue that politicians have very little incentives to devote attention to the preferences of the median voters: only a small proportion of the mass public pays attention to, know about, and participate in politics; and those who do are more likely to be partisans than the typical median voter (Hibbing and Theiss-Morse 2002; Delli Carpini and Keeter 1996; de Vreese and Boomgaarden 2006).^5^ Nevertheless, existing empirical evidence still gives some credit to the Downsian logic. An extensive literature on cue-taking argues that even the least informed and attentive public often draws on multiple sources of information to make decisions about politics, also keeping politicians in check (Lau and Redlawsk 2006; Lupia, McCubbins, and Arthur 1998). In fact, in their exhaustive work on policy responsiveness in the United States and Canada, Soroka and Wlezien (2009) find “roughly the same degree [of policy responsiveness] across groups. In most cases, representation is neither markedly better nor markedly worse when we look solely at certain groups” (p. 165).^6^ Hence, given the conflicting arguments and evidence, it is pertinent to test whether a Downsian logic is still in place.Amain testable hypothesis that derives from the argument is that **(H3) changes in attention allocation by the *general public* predict changes in issue attention by members of Congress.**

Other scholars, however, disagreewith this premise as being too optimistic about the public’s agendasetting role. Instead of responding to the median voter or the general public, some believe members of Congress have incentives to be mostly responsive to attentive voters. Studies of opinion formation show that most voters do not follow day-to-day politics (Hibbing and Theiss-Morse 2002) and that many do not have clear issue priorities nor policy preferences (Converse 2006). Nevertheless, this is not the case for all citizens. Some attentive voters care a great deal about the political world, and according to theoretical models such as Katz and Lazarsfeld (1955)’s “two-step communication flow” and Page and Shapiro (1992)’s “rational public,” these attentive voters have the potential to influence the issue priorities and preferences of less attentive citizens. This type of logic leads congressional scholars such as Arnold (1990) and Aldrich (1995) to argue that members of Congress should be particularly concerned about the issues to which attentive voters pay attention. A testable hypothesis that derives from this logic is that **(H4) changes in attention allocation by *attentive publics* predict allocation changes by members of Congress.**

Another group of researchers proposes a third alternative: legislators should be mostly interested in responding to core party supporters. They have issue priorities that are easier to distinguish and represent (Wright 1989), they play a very active role in nomination processes (Bawn et al. 2012), their support is crucial to win not only primaries (Fenno 1978; Gerber and Morton 1998) but also general elections (Holbrook andMcClurg 2005), and the priorities of policyoriented members are more likely to align with theirs (Egan 2013; Kastellec et al. 2015). Some empirical research finds that in fact legislators are more likely to represent the policy preferences of their supporters (Clinton 2006; Kastellec et al. 2015; Neundorf and Adams 2018; Shapiro et al. 1990), but no research yet exists showing whether that is the case for issue attention allocation. From this model, however, we can derive that **(H5) changes in attention allocation by *party supporters* predict allocation changes by members of Congress.**

As a final theoretical consideration, scholars argue (and find) that the more salient an issue becomes in the eyes of the public, the larger the degree of political responsiveness we should expect (Burstein 2003; Canes-Wrone and Shotts 2004; Jones 2004; Soroka and Wlezien 2009; Sulkin 2005): “the public importance of policy domains may tell us a lot about policy makers’ responsiveness. There is good reason, after all, to expect policymakers to reflect the importance of the different domains because of possible electoral consequences” (Wlezien 2004, 7). If politicians aim to be responsive to a certain group of the public, they should be interested in reacting to shifts in attention involving issues that are particularly salient to that group. In other words, if members of Congress are mainly responsive to their party supporters (or to the attentive or general public), they should be more likely to react to shifts in attention by party supporters on issues that take on 10% rather than 1% of their supporters’ discussion. Hence, building on this literature, if the previous responsiveness models apply, we should expect that **(H6) to the extent that particular issues are more salient among the general public** (according to the Downsian model), **attentive citizens** (according to the Attentive model), **and party supporters** (according to the Supporter model), **these publics’ priorities will more strongly predict the politicians’ agenda.**^7^

## THE MECHANISMS OF ISSUE RESPONSIVENESS

Here, we use messages sent on Twitter by politicians and the public as measures of these individuals’ issue priorities, allowing us to evaluate the extent to which reciprocal relationships exist among these priorities. Previous research has suggested several mechanisms by which politicians might become informed about the public’s priorities, and vice versa. In particular, this literature puts forward a set of mechanisms through which the issue priorities of politicians and the public can directly lead the priorities of the other, as well as ways in which the mass media can channel these reciprocal dynamics.

One mechanism by which politicians and citizens learn about one another’s priorities is direct interaction and communication between lawmakers and their constituents, which include town halls, “lobby days” organized by interest groups and offline and online correspondence. For example, about24%ofAmericans report having written a letter to a public representative (Schlozman, Verba, and Brady 2012). Surveys indicate that politicians pay some attention to social media messages from constituents as well, although these messages are not weighed as heavily as other constituent communications (Chen, Lee, and Marble 2018). Another way that politicians learn about citizens’ priorities is by tracking public opinion. Polling organizations, such as Gallup and the Pew Research Center, regularly release polls revealing the public’s issue priorities. Political parties, campaign staff, and government agencies run their own polls to assess the issues that are of interest to the public (Jacobs and Shapiro 2000). Modern practices also include using dashboards to track the issues that are mostly discussed by citizens in social media (Webster and Ksiazek 2012).

In this study, we focus on what is arguably the primary mechanism by which politicians and the public learn about each other’s issue priorities: the news media. First, the media’s powerful role in this regard derives from the fact that lawmakers are incentivized to expend effort to generate media coverage of their priorities, while at the same time media outlets are incentivized to cover issues that resonate with their audiences’ priorities. A wide range of factors determine media content, including assigned “beats,” journalistic practices, and the occurrence of newsworthy events (Graber 1997; Shoemaker and Reese 1996). Among these factors, audience preferences and political institutions play a very important role, ensuring that media covers issues that are of interest to both the public and politicians. And second, the media’s substantive role derives from its ability to drive both public and political attention (McCombs and Shaw 1972; Zaller 1992).

The media is responsive to political elites in part because political institutions represent an important source of constant newsworthy information (Shoemaker and Reese 1996). Media outlets regularly appoint correspondents to institutions such as the White House and Congress, ensuring that major issues discussed in these political venues achieve media attention. Media outlets are responsive to public demands in part because of market pressures: particularly in a context in which most outlets face economic hardships, discussing the issues that are of interest to the public increases their chances of getting the readers and viewers needed to generate profits. More than ever, media outlets today have a wide range of instruments in their hands to measure, and respond to, the issues in which the public is interested (including tracking social media attention) (Anand Peterson 2000; Webster and Ksiazek 2012). In addition, public sentiment is often reflected by newsworthy political events in themselves, including election results, strikes, and demonstrations (Gitlin 1980).To be sure, neither the public nor politicians have exclusive control over the agenda, which is frequently set by external unexpected shocks (such as natural disasters) (Birkland 1998) and recurrent events (and expiring statutory provisions) (Adler and Wilkerson 2012) that simultaneously affect the attention distribution of politicians but also of the media and the public.

There is clear evidence showing that not only politicians and the public lead media attention but also that the media can drive political agendas and public opinion (Soroka 2002; Walgrave, Stuart, and Nuytemans 2008; Walgrave and Van Aelst 2006). The media “construct” and highlight problems for politicians to solve, and they increase the salience of issues that voters might consider relevant, which reelection-seeking politicians should address in other to please them (Wouters and Walgrave 2017). Moreover, a long-standing literature also shows that the issues covered in the media are very likely to lead public opinion and preferences (Boydstun 2013; Iyengar and Kinder 2010; McCombs and Shaw 1972; Zaller 1992).

Exploring all the mechanisms through which groups of the public and politicians influence the agenda of the other is outside the scope of this study. Nevertheless, given that research points to mass media as playing a crucial agenda setting role, in our analysis we will control for potential media effects as well as explore the extent to which mass media coverage favors particular responsiveness models.

## DATA

### Members of Congress on Twitter

To test our hypotheses, we use tweets sent by members of the 113th House and Senate of the US Congress (2013–14). Twitter use in Congress has increased steadily over the past years (Chi and Yang 2011; Evans, Cordova, and Sipole 2014; Golbeck, Grimes, and Rogers 2010; Shapiro, Hemphill, and Otterbacher 2012). Ninety-five percent of legislators that served in the 113th Congress had active Twitter accounts, sending a total of 651,116 messages (excluding retweets), about 900 tweets per day.^8^

Golbeck, Grimes, and Rogers (2010) argue that members of Congress use Twitter primarily to advertise their policy positions and to provide information about their activities. However, more recent studies have shown that the platform can also be a tool for members of Congress to be responsive to their constituents, exercise control of the political agenda, express party loyalty, engage in partisan taunting, and report on their constituency service (Hemphill, Otterbacher, and Shapiro 2013; Evans, Cordova, and Sipole 2014; Russell 2018b, 2018a). Moreover, research indicates that the topics discussed in tweets are a fair representation of the legislators’ overall expressed agenda: there is a very high correlation between the issues they discuss on social media and their press releases, for example (Casas and Morar 2015).

### Citizens on Twitter

In addition to tweets sent by members of Congress, we also collected tweets sent by different samples of Twitter users. These allow us to test our hypotheses (H_3, 4, 5, 6_) regarding the part of the public whose shifts in attention politicians are more likely to follow. We consider four samples of Twitter users:

1. **General Public:** includes about 25,000 Twitter users, sampled by generating random numeric user IDs, then checking whether the users existed, and then checking whether the users resided in the United States.^9^
2. **Attentive Public:** a randomly generated sample of Twitter users that follow at least one of five major media outlets in the United States (CNN, Wall Street Journal, New York Times, Fox News, and MSNBC). We apply a geographic restriction based on the time zone on users’ profiles, which is available for most users. In particular, we exclude users whose time zone indicates they are likely to be located outside the United States. We also filter based on activity: only users who have ever sent 100 tweets or more are included. After applying these filters, the final sample size is 10,000 users.
3. **Republican Supporters:** a random sample of 10,000 Twitter users who follow three or more Republican members of Congress and no Democrat in Congress. The same geographic and activity filters as in the attentive public sample are applied here. In Online Appendix F, we demonstrate why this sampling method is able to select party supporters.
4. **Democratic Supporters:** a random sample of 10,000 Twitter users who follow three or more Democratic members of Congress and no Republican in Congress. The same geographic and activity filters as in the sample of Republican supporters apply.

After identifying these four samples, we then collected all the tweets they sent during our period of analysis (January 2013 to December 2014) using Twitter’s REST API.^10^ The final number of users and tweets in each group is available in Table 1. Retweets are excluded from our sample to avoid inflating the correlations we observe between politicians and the public regarding the issues they discuss.

**TABLE 1:** Description of the Tweets in the Dataset

Group	*N*	Avg	Min	Max	Tweets
House Republicans	238	1,215	70	8,857	267,311
House Democrats	207	1,177	113	5,993	222,491
Senate Republicans	46	1,532	73	6,627	67,412
Senate Democrats	56	1,616	150	10,736	87,307
Random sample	25k	465	1	8,926	11,316,396
Informed public	10k	948	100	5,861	9,487,382
Republican supporters	10k	1,091	100	8,804	10,911,813
Democratic supporters	10k	1,306	100	5,122	13,058,947
Media outlets	36	7,803	8	15,858	273,121

*Note:* Period of analysis: January 1, 2013, to December 31, 2014.Ncorresponds to the number of Twitter accounts in each sample. *Avg, Min*, and *Max* correspond to the average, minimum, and maximum number of tweets, respectively, sent by individual users in each group during the whole period of analysis. *Tweets* corresponds to the total number of tweets sent by all users in each group during the period of analysis.

### Media

As discussed previously, it may well be that both public and political issue agendas are led by the mass media (Gerber, Dean, and Bergan 2009; Habel 2012; King, Schneer, and White 2017; Ladd and Lenz 2009), particularly soon social media (Feezell 2018).To account for this possibility, we also collected tweets froma sample of media outlets and use them to control for media effects. In particular, we collected all tweets sent over the same time period from the Twitter accounts of the 36 largest media outlets in the United States (print, broadcast, online), as identified by the Pew Research Center.

## MEASURING ATTENTION TO POLITICAL ISSUES WITH TOPIC MODELS

Our purpose in this paper is to characterize the different issues that members of Congress, ordinary citizens, and media outlets discussed on Twitter, and how their importance varies over time and across groups defined by their partisanship and political interest. To extract these categories, we estimate a probabilistic model of word occurrences in documents called an LDA (Blei, Ng, and Jordan 2003), which belongs to a general category of latent variable models that infer topics from documents using a “bag-of-words” approach.

As we explain in greater detail in Online Appendix G.1, this method treats each document as a random mixture over latent topics, and each topic as a probability distribution over tokens. In our analysis, tokens are *n*-grams (combinations of one and two words). Our definition of “document” is the aggregated total of tweets sent by members of Congress each day, by party and chamber.^11^

The alternative to using an unsupervised topic model would be for the analyst to choose the topics and then build a supervised classifier predicting them. Despite the existence of well-known categories of political issues,^12^ training an accurate classifier would be an incredibly arduous task, given the large number of categories, making unsupervised models a preferable option. However, as we show in Online Appendix E.2, it is possible to map the topics derived from the data to an existing classification of political topics—the topics used in Jones and Baumgartner (2004)—with similar results.^13^ And as we demonstrate in Online Appendix G.3, the topics generated by the model pass standard tests of both predictive and semantic validity (Quinn et al. 2010).

Note two additional features of our analysis. First, we fit the model at first only for members of Congress (instead of fitting it to the messages sent by all groups) to increase the likelihood of discovering topics that were politically salient during the 113thCongress, and then use the estimated parameters to compute the posterior topic distributions for citizens and media outlets, also aggregated by day, based on their observed words. However, a replication of the results based on an LDA model fit to the tweets of politicians, the media, and the public leads to similar conclusions (see Online Appendix E.4). Second, in our estimation we assume that topic distributions are independent over time, and that the number of topics and the content of each topic is constant over time.

Wefix the number of topics to *K* = 100 after exploring a wide range of values by running 10-fold cross-validations and computing common goodness-of-fit measures (Chang et al. 2009) (see Online Appendix G.2 for a detailed description of how we chose *K*).

In general, we find that most of the 100 resulting topics can be easily labeled. However, not all of the mare political in nature: for example, we find topics about anniversaries and celebrations (Valentine’s Day, Flag Day, Constitution Day, Thanksgiving, etc.). Because we are not interested in these topics, in our analysis we will only include political issues, of which 53 were identified. 14 After reviewing their content, we noted that some topics that referred to a single issue were classified as different topics because distinct words were being used by different groups when talking about the same issue. For example, we found separate topics for Republican and Democratic members of Congress discussing the 2013 Government Shutdown. This may influence our results by overestimating how often parties in Congress respond to their supporters. To avoid this potential source of bias, we decided to merge some topics and focus our analysis on 46 political issues. Table 2 displays the list of all these topics we have classified as political issues.

**TABLE 2:** List of Political Issues

Topic Number	Label	Topic number	Label
3	Investigation of Benghazi attack	50	Climate change
7	100 days of #BringBackOurGirls campaign	51	Lame duck congress
9	Gender wage gap	53	Minimum wage
12	Republican issues Spring 2013	58	Affordable Care Act
14	Marriage equality	62	Border crisis in Texas
15	Gun violence	63	Obamacare (employer mandate)
16	Abortion (pro-life)	64	FAA furloughs cause flight delays
18	Veteran affairs delays scandal	66	Malaysia Airlines crash in Ukraine
20	NSA surveillance scandal	67	Comprehensive immigration reform
23	#BringBackOurGirls campaign	70	#MiddleClassFirst campaign
28	Employment Non-Discrimination Act	75	Military Justice Improvement Act
32	Islamic state	81	Poverty (SNAP program)
33	Use of military force in Syria	83	Twenty-first century cures initiative
36	Ebola	85	Unemployment insurance
37	Social security	88	IRS scandal
39	Keystone XL pipeline	89	Obamacare (website and implementation)
41	Immigration (border security)	93	Jobs bills omnibus
43	Executive action on immigration	96	Violence Against Women Act
46	Unemployment numbers reports	97	Protests in Ukraine and Venezuela
47	Paul Ryan budget proposal	99	CIA detentions and interrogations report
48	Black history month	100	#ObamacareInThreeWords campaign
(101)	Student debt	(102)	Hobby lobby supreme court decision
(103)	Budget discussion	(104)	2013 government shutdown

*Note:* The topic number in parentheses indicate issues that have been created ad hoc by merging very similar topics from the topic model.

## RESULTS

### Issue Attention Congruence

The key substantive question we want to answer is whether the distribution of topics discussed by members of Congress leads or follows that of their constituents, and vice versa. Are members following their constituents? And if so, are they following particular types of constituents?

Similar to previous studies on the issue, we start by examining simple congruence in the way members of Congress and citizens allocate attention to the 46political issues we identified. In this issue congruence framework, a correlation between the public and the political agenda is a necessary condition for political responsiveness to be present. Table 3 displays Pearson correlation coefficients, indicating how similar the issue distribution of Democratic and Republican supporters, attentive publics, and the general public are to the expressed agenda of Republicans and Democrats in Congress over the two-year period studied. Higher coefficients indicate that groups tend to discuss the same issues.

**TABLE 3:** Correlation in Issue Attention Between Members of Congress and Groups of the Public and the Media Over 46 Political Issues

Group	Democrats in Congress	Republicans in Congress
Democratic supporters	0.69	0.51
Republican supporters	0.41	0.77
Attentive public	0.49	0.52
General public	0.38	0.34
Media	0.52	0.63

These initial results show potential for corroborating the presence of political responsiveness at the issue attention level, and they seem to indicate that some responsiveness models have a stronger explanatory power than others. In particular, these results provide stronger support for the Supporter and, to a lesser extent, the Attentive models, than for the Downsian argument. There is a positive, and in some cases large, correlation between the agenda of members of Congress and the issues discussed by their constituents. Nevertheless, when paying attention to the coefficients for specific groups, we observe the highest correlations to be between members and their party supporters (0.69 for Democrats and 0.77 for Republicans) and between members and the attentive public (0.49 for Democrats and 0.52 for Republicans). The correlation between the expressed agenda of legislators and the attention allocation of supporters of the other party is much lower (a 0.41 correlation between Democratic members and Republican supporters, and 0.51 between Republican members and Democratic supporters). We observe the lowest correlation coefficients when comparing the agenda of lawmakers and the issues the general public discuss. As expected, the issue attention distribution of the media is also highly correlated to the agenda of members of Congress, emphasizing mass media’s agenda-setting and mediating role.

**FIGURE 1:**
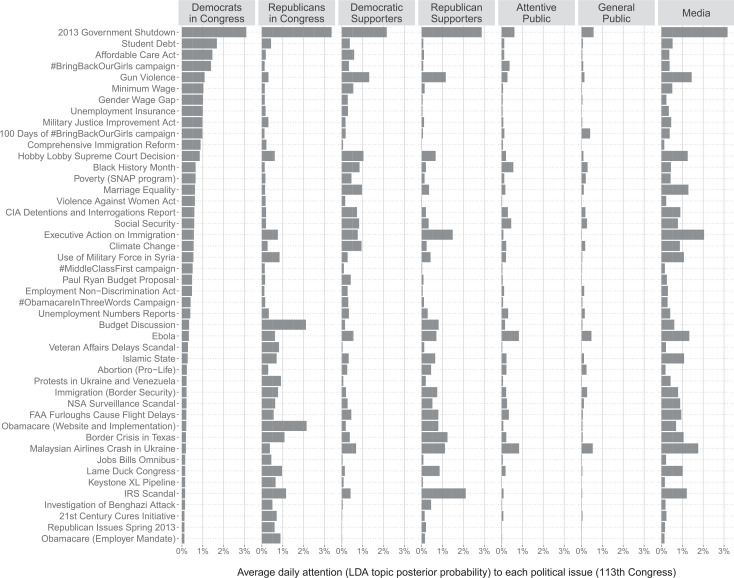
Average Issue Attention by Groups of Politicians, the Public, and the Media *Note:* Attention is represented as daily posterior LDA topic probabilities expressed in percentages. These are percentages based on all 100 topics of the LDA model.

In Figure 1, we provide information about the average daily attention that each party in Congress, each public group, and the media paid to the political issues under study. This figure provides a more detailed understanding of the agenda level correlations we observe in Table 3 and some potential reasons as to why we observe a particularly strong relationship between the issue agenda of members of Congress and the attention distribution of their party supporters. We see, for example, how Democrats in Congress and Democratic supporters paid much more general attention to the Affordable Care Act (row 3 of figure 1) and Marriage Equality (row 15) than did Republicans, whereas Republicans in Congress and Republican supporters paid more attention than did Democrats to the (troubled) release and implementation of the ACA website (11th to the last row), its employer mandate clause (last row), and to the Border Crisis in Texas (10th to the last row). The attentive public, and especially the general public, paid less attention not only to these issues but also to all political issues in general. (See Online Appendix G.4 for further discussion of these results.)

### Who Leads? Who Follows?

The previous correlations and percentages, however, are not sufficient evidence to conclude that members of Congress lead the issue attention distribution of their constituents nor to adjudicate between the competing Downsian, Attentive, and Supporter models. Here, we take advantage of the time series nature of our dataset to establish who puts issues on the agenda first by estimating a VAR models with topic-fixed effects. These models are well-suited to capture the relationship between endogenous variables (Freeman, Williams, and Lin 1989; Sims 1980) and have been used in previous political science studies with similar objectives (Edwards andWood^1999^; Enders and Sandler 1993; Wood and Peake 1998).^15^

In our VAR model, we have a set of stationary time series Yi representing the proportion of daily attention each of our groups *i*^16^ paid to each topic *j* in day *t* of the 113th Congress.^17^ The values of these random variables range from 0 to 1 but neither of the extreme values are present (0< *Y_ijt_*<1).Their distributions are right skewed, with few days of very high issue attention and much lower attention during the rest of the two-year period. Wefollow a common practice in time series analysis of skewed proportions (Wallis 1987) and model the log odds *Z_i_* of the described series *Y_i_* instead of the raw proportions.

We then express the autoregressive and endogenous relationship of these variables as a system of equations in which each variable *Z_i_* is a function of its previous lags plus the lags of the other variables. Given that there are no time restrictions when it comes to posting messages on Twitter, we would theoretically expect members of Congress to follow changes in public issue attention quite rapidly. However, to account for the potential of longer-term decay we use a seven-lag structure.^18^ The final model can be & formally expressed as follows:

$$\begin{array}{l} Z=\log\left(\frac{Y}{1-Y}\right)\\ Z_{i,j,t}=\alpha_{j}+\sum_{i}\sum_{p=1}^{7}\beta_{i,p}Z_{i,j,t-p}+\varepsilon_{i,j,t} \end{array}$$

Note that given the issue-fixed effects structure of the model (*α_j_*), we are assuming that the estimates of interest are constant across issues. Although this is an inaccurate assumption, it is a useful one for what we intend to accomplish here. It allows us to estimate how much on average we should expect changes in issue attention by a given group to predict subsequent attention allocation of the other groups.^19^

**FIGURE 2:**
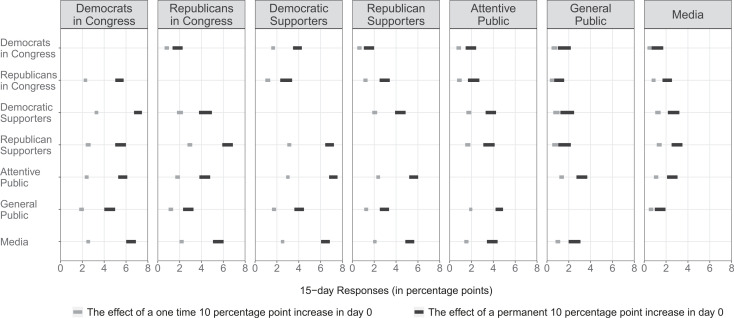
15 Day Cumulative IRFs: Predicted Issue Responsiveness Across Groups *Note:* The coefficients (with 95% confidence intervals) indicate (in percentage points) how much more cumulative attention the groups in the panel titles paid to a given issue as a result of the groups in the y-axis increasing the attention to the same issue by 10 percentage points once (in gray) and permanently (in black) 15 days ago.

The results of the estimated VAR model can be best expressed using cumulative impulse response functions (IRFs). These cumulative IRFs indicate how an x-unit increase in attention to a given topic by a group predicts the cumulative attention that other actors dedicate to the same topic over time. Cumulative IRFs can be calculated for a varying number of subsequent days. We calculate and report in Figure 2 two different types of IRFs for a 15-day period.^20^ In both cases, we assume that at day 0 none of the groups is paying attention to a given issue *j*. First, we want to explore the effect of brief changes in attention and we calculate how a 10 percentage point increase in attention to an issue by each group (going from 0% to 10% of attention in day 0) affects future issue attention by the other groups. We are also interested in the effect of attention changes that last longer and calculate how a permanent attention change to a given issue from 0% to 10% by one group affects the attention of the others.^21^ Each single panel in Figure 2 shows how much more cumulative attention to the issue the group in the panel title is predicted to pay after a one-time (in gray) and permanent (in black) 10 percentage point increase by the groups along the y-axis (the row groups) 15 days ago. The predicted responses (95% confidence interval lines) are expressed in percentage points (0–100 scale).Most one-time effects (gray coefficients) range from 0 to 4. We believe these are meaningful and substantive responses. As weknowfrom the long-standing literature on agenda setting, politics is often a fight for attention and simply getting an issue into the agenda is extremely difficult (Jones and Baumgartner 2005; Schattschneider 1975). Moreover, dynamics of attention often follow nonlinear functions with tipping points, where small amounts of additional attention around the tipping point can have large political implications (Baumgartner and Jones 1993; Kingdon 2013).

The results in Figure 2 corroborate the first two expectations in regard to the ability of members of Congress and the public to predict each other’s issue attention. Politicians from both parties (first two rows in all the panels in Figure 2) are able to predict the attention distribution of the public (H_2_). Specifically, they are able to lead the issue attention of party supporters and attentive publics, although both parties lead the issue attention of their own supporters than supporters of the opposing party. Andwe see in the far right panel that the issues prioritized by both parties appear to be very poor predictors of the issue attention of the general public.

We also find strong evidence supporting a political responsiveness dynamic (H_1_): we see changes in issue attention by citizens to be positive predictors of the issues members of Congress discuss, and we also see these effects to always be of a larger magnitude than the ability of members of Congress to lead the agenda of the public. The ability of Republican supporters to set political issue agendas represents the most extreme case. This group of the public is predicted to increase the cumulative amount of attention to an issue only by 0.75 and 1.25 percentage points 15 days after a 10-point increase in attention by Democrats and Republicans in Congress, respectively (first and second gray estimates from the top in the fourth panel from the right). The changes in the opposite direction, however, are two to three times larger: both Democrats and Republicans in Congress are predicted to increase their cumulative attention by about 3 percentage points (Republican supporters’ gray estimates in the two most left panels).

**FIGURE 3:**
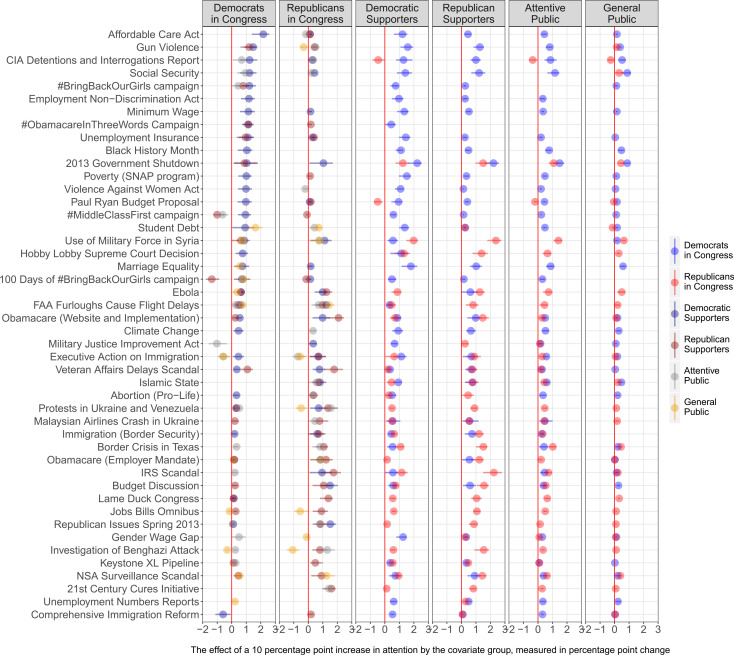
Politicians’ Ability to Set Public Agendas versus the Ability of the Public to Influence Political Agendas *Note:* The coefficients (with 95% confidence intervals) indicate (in percentage points) how much more cumulative attention the groups in the panel titles paid to a given issue as a result of the groups in the y-axis increasing the attention to the same issue by 10 percentage points (in black) 15 days ago. The gray coefficients indicate the vice versa effect.

These differential effects can be better appreciated in Figure 3, where we rearrange the one-time attention changes estimates (gray responses) from Figure 2 to more easily compare who has the largest ability to lead the issue agenda of the other, members of Congress or the public. However, there is an additional factor we need to take into consideration when interpreting these results. As we observed in Figure 5 and Table A.6, politicians devote more attention to political issues than their party supporters (and much more than the attentive and general public). This means that although an increase in attention of the same size has a larger effect when it goes from the public to politicians, we are more likely to observe members of Congress, rather than the public, to make large shifts in political attention.

Our results also provide strong evidence in favor of the Supporter model of responsiveness (H5). If we focus only on the variables predicting the agenda of members of Congress the most (two left panels in Figure 2), we observe that the strongest predictors of a positive attention change by lawmakers is a change of attention by their own party supporters. The VAR model predicts Democrats in Congress to go from paying no attention to an issue to dedicating a cumulative attention of approximately 3% as a result of a one-time 10 point attention shift by Democratic supporters, and 7% as a result of permanent 10 point change by their party supporters (Democratic supporter estimates in the left panel). We see Republicans in Congress respond similarly to changes in attention by their own supporters (Republican supporter estimates in the second panel from the left). All the other IRFs for the one-time and permanent attention shocks are of smaller magnitude.

We also find some support for the Attentive model (H_4_). For example, after a one-time and a permanent 10 percentage point change in attention by the attentive public, Democratic members of Congress are predicted to increase their cumulative attention by 2.25 and 5.75 percentage points, respectively, and Republican policy makers by about 1.75 and 4.25. If we treat the supporters of the other party also as an attentive public (they follow not one but at least three members of Congress in Twitter), we observe a similar pattern. Changes in attention by Democratic Supporters are also predicted to have a positive effect of 2 and 4.25 points on Republican members, and changes by Republican Supporters are predicted to increase the cumulative attention of Democratic lawmakers by 2.5 and 5.5 points. However, the estimated effects are of smaller size than the effects we observed in favor of the Supporter model.

Finally, the results show weak support for the Downsian model (H3). Democratic members of Congress are only predicted to increase their cumulative attention to an issue by 2 and 4.5 percentage points after a one-time and a permanent 10 point increase of attention by the general public. The Republican members’ response is expected to be even lower—their cumulative attention increases only by 1.5 and 3 percentage points. This means that among the different groups of the public, the General Public has the lowest ability to lead the agenda of members of Congress. The effect of a permanent increase of attention by the general public (black general public estimates in the two left panels) is of a similar magnitude to a one-time attention increase by party supporters (Democratic supporter gray estimate in the left panel and Republican supporter gray coefficient in the second panel from the left). Moreover, given the low attention the general public pays to politics, attention shifts of this magnitude (10 percentage points) are unlikely to take place. Nevertheless, when comparing the results of fitting the same model only to data from 2013 with data only from 2014, we interestingly find that politicians are slightly more responsive to the general public during election year (2014) than during a nonelection year (although even during an election year politicians are also more likely to follow shifts in attention by party supporters and the attentive public).^22^

Overall, the results show that politicians are more likely to follow changes in issue attention distribution by their own party supporters than to attentive voters, and that they rarely follow the issue priorities of the general public.

### Responsiveness and Issue Relevance

If members of Congress have an interest in being responsive to specific groups of constituents, then we expect that (H_6_) they should be particularly interested in responding to changes in attention involving issues that are salient to these groups. To test this hypothesis, we first need to estimate by how much each group led the attention that all other groups paid to each separate political topic. To do so, we relax our assumption that the ability of one group to lead the agenda of the others is constant across issues, and we model the data in a different way. In the previous model, we included topic-fixed effects (*α_j_*). In this section, we instead estimate 46 separate VAR models, one for each political issue. We include the same endogenous variables into the model, again apply a logit transformation to all time series and use the same seven-lag structure. Then, for each of the VAR issue models, we calculate 15-day cumulative IRFs capturing how a one-time 10 percentage point increase in attention by a specific group predicts the attention of the other groups.

**FIGURE 4:**
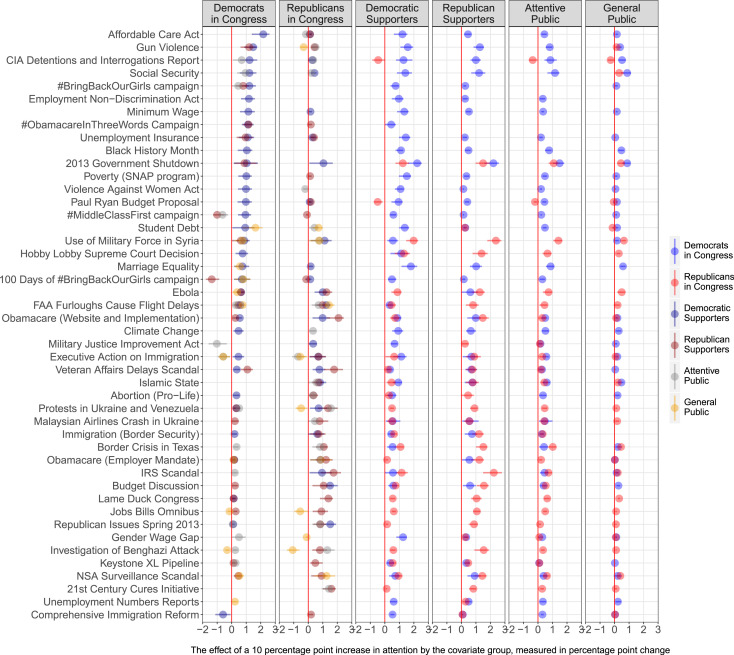
Predicted Issue Responsiveness Across Issues and Groups (15 Day IRFs) *Note:* The coefficients (with95%confidence intervals) indicate (in percentage points) how much more/less cumulative attention the groups in the panel titles paid to the issue in the y-axis as a result of a group (identified by the color) increasing the cumulative attention to the same issue by 10 percentage points 15 days ago. Only coefficients not crossing zero have been included. The two left-most panels show the influence of the public on Members of Congress. The four right-most panels show the influence of Democratic and Republican members of Congress on the public. Versions of this figure that also show the coefficients crossing zero are available in Online Appendix C.

Figure 4 shows the results (15-day IRFs) for each of these 46 VAR models. Each panel reports how the groups in the panel titles are predicted to respond to changes in attention by the other groups: the circles represent the predicted effects (with lines representing a 95% confidence intervals), the colors of the circles show the group to which they are reacting, and the labels on the y-axis (the row labels) indicate the specific issue. To avoid overcrowding the plot, in the two left-most panels we only show the ability of the public groups to lead the expressed agenda of Democrats and Republicans in Congress, and in the four panels on the right we show the reverse effects, the ability of members of Congress to lead public issue attention. We only include the predicted effects for issues where the confidence intervals do not cross zero.^23^

**FIGURE 5:**
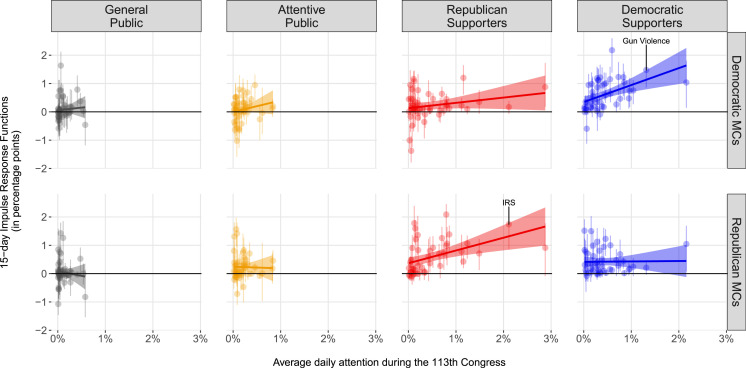
Correlation Between Public Issue Relevance and the Ability of the Public to Set Political Agendas *Note:* The x-axis indicates the average attention the groups in the top panel titles paid to each political topic during the 113th Congress. The *y*-axis indicates how much more/less cumulative attention Democrats (top four panels) and Republicans in Congress (bottom four panels) paid to these topics as a result of the groups in the top panel titles increasing the attention to the topic by10percentagepoints15days ago. Each dot represents a different political issue and the lines around the dots represent95%confidence intervals. Rowsare sorted by the largest effect of Democrats in Congress (left panel).

The issues are sorted based on the predicted impact of Democratic Supporters on Democrats in Congress: from the issues with the largest estimated impact to the issues with the smallest. Among the top rows, we find issues such as healthcare reform (“Affordable Care Act,” row 1), gun violence (row 2), and minimum wage (row 7). A one-time 10-percentage-point increase on these issues by Democratic supporters is predicted to increase the cumulative attention that Democratic members of Congress pay to them by 2, 1.5, and 1 percentage points.

In the second panel from the left, we see the issues in which Republican supporters more strongly led the agenda of Republicans in Congress by looking at the red circles. The discussions around the IRS scandal (row 35) that took place around mid-2013 and the implementation of the Affordable Care Act and its website problems (row 23) were the issues on which Republican members of congress seemed to follow their supporters the most. In both cases, a one-time 10 percentage point shift is predicted to translate into an increase of cumulative attention of about 2 percentage points 15 days later. The effects represented by gray circles in the leftmost panel indicate that social security (row 4) is the issue on which the attentive public was able to lead the discussion of Democratic members the most, and in the second panel, we see that they particularly led the attention that Republican members paid to the protests in Ukraine and Venezuela (row 30). The effects represented by orange circles in the two left panels indicate that discussions by Democratic and Republican members of congress on student debt (row 16) and the use of military force in Syria (row 17), respectively, were among the ones that the general public was able to positively lead the most.

To test the last of our hypothesis, that members of Congress will be more likely to follow shifts in attention on issues to which constituents pay more attention (H_6_), we build a measure of group issue relevance by calculating the average daily attention each group paid to each topic during the 113th Congress (these averages are displayed in Figure 1). By taking the average, we intend to focus less on how much attention a group paid to a given issue at a particular point in time and to capture instead how important the issue was for that specific group in general.

With this measure of average attention and the estimates from Figure 4 in hand, we can now move to a direct test of H_6_ by examining correlations between the two. Accordingly, in Figure 5 we plot on the x-axis the average daily attention paid to each issue by each group of the public (see panel titles). In the y-axis, we plot the cumulative attention members of Congress are predicted to pay to each issue as result of the groups in the panel title increasing their attention by 10 percentage points 15 days ago (15 day cumulative IRFs). Each dot is a single predicted response and the lines around them represent 95% confidence intervals. The four top panels show the predicted response of Democratic members of Congress, whereas the bottom ones illustrate the predicted reaction of Republican lawmakers.

We find support for the issue relevance hypothesis (H_6_) only as it relates to the Supporter model. In the top right panel, we observe that changes in attention by Democratic supporters have a larger effect on the agenda of Democrats in Congress when they involve issues Democratic supporters deem relevant (such as gun violence). In the second from the right bottom panel, we also observe a similar pattern for Republicans, with Republican supporters being more likely to lead the expressed agenda of Republicans in Congress on issues that are important to them (such as the discussion around the Internal Revenue Service—IRS). Thus, members of congress appear to behave as if they are more likely to pay attention to the views of their supporters on issues their supporters care more about than on issues they care less.

In Figure 5, we see no support for the issue relevance hypothesis as it relates to the Attentive and Downsian models. On average, Democrats in Congress are more likely to follow changes in attention by the attentive public (including supporters of the other party) on issues that are relevant to these groups (two middle panels on the top).However, these positive correlations are of a very small magnitude. For Republicans in Congress, we do not even see a positive correlation between how much they follow shifts in attention on particular topics and the average attention the attentive public and supporters of the other party paid to the issues (flat yellow and blue lines at the bottom). Finally, we observe no correlation between how much the general public leads politicians’ agenda and the amount of average attention the general public devotes to any given issue. Overall, these results validate the strong findings from Figure 2 in favor of the Supporter model, as well as the lack of evidence in support of the Downsian model of responsiveness.

### The Role of the Media

We now turn to an evaluation of the role played by news media in mediating the dynamics identified in this paper. Our data and methods allow us to examine in detail whether the mass media is equally likely to lead, and be led by, politicians and the public—or whether, in contrast, the media strengthens the voice of some groups and increases their ability to lead political agendas.

As a first cut, Table 4 confirms previous work indicating that media coverage reflects both politicians’ and the public’s issue priorities. The table displays correlations between the distribution of attention to issues by the media and that of the other groups under study. We find a particularly strong relationship between media issue attention and the issue attention of politically engaged Americans—that is, party supporters and the attentive public. Less substantial, but still very strong, relationships exist between the issue attention of mass media and that of members of Congress and the general public.

**TABLE 4:** Correlation in Issue Attention Between Media Outlets and the Other Groups of Analysis

Group	Media
Democrats in Congress	0.52
Republicans in Congress	0.63
Attentive public	0.74
Democratic supporters	0.79
Republican supporters	0.79
General public	0.55

**FIGURE 6:**
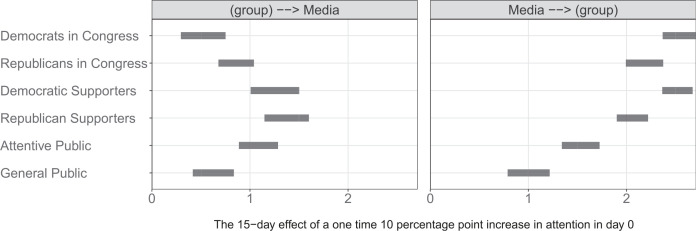
Predicted Media Effects *Note:* The effects (with 95%) in the left panel indicate how much the media outlets increased their attention to a given issue (in percentage points) 15 days after the groups in the y-axis increased their attention to the same issue by 10 percentage points. The coefficients in the right panel indicates the vice versa effects, how much the groups in the y-axis increase their attention to an issue 15 days after the media increased the attention to the same issue by 10 percentage points.

These correlations, of course, do not provide information about the directions of these relationships. For a clearer picture of the role of the media, we take a closer look at the IRF coefficient estimates regarding the media originally displayed in the final column and bottom row of Figure 2, plotting them in more detail in Figure 6. In this figure’s left panel, media coverage is the dependent variable. Plotted here are the estimated impacts of changes in the attention to issues given by politicians and the public on the attention given to these issues by media. Here, we observe that demand-side forces (namely, the priorities of the most politically engaged Americans) are stronger predictors of what the media covers than supply-side forces (i.e., the priorities of members of Congress). These results corroborate the argument that media outlets are particularly likely to follow shifts in attention by the public due to market pressures (Anand and Peterson 2000; Webster and Ksiazek 2012) and suggest that the effort expended by lawmakers to raise the salience of their favored issues is relatively less influential.

In the right panel of Figure 6, media coverage is the independent variable. This panel displays the estimated impacts of changes in media issue attention on the attention given to these issues by politicians and the public. Shifts in issue attention by media outlets have the strongest impact on the issue agendas of members of Congress and of party supporters. Notably, in each case, the power of shifts in media attention to predict subsequent shifts in attention among all audiences is greater than the reverse, confirming that media outlets play a crucial role in leading political attention (Soroka 2002; Walgrave, Stuart, and Nuytemans 2008; Walgrave and Van Aelst 2006). Finally, the media effects in Figure 6 suggest that news media contribute to the promotion of a Supporter responsiveness model. Not all groups of the public are equally likely to lead the issues covered by media outlets. The “voice” of Democratic and Republican supporters is stronger than the voice of the attentive citizens and the general public. This is particularly relevant given that, as we observe in the right panel in Figure 6, the issues covered by the media are strong predictors of the subsequently expressed issue agenda of members of Congress.

## DISCUSSION AND CONCLUSIONS

It is well known in American politics that politicians and the public tend to pay attention to the same political issues (Jones and Baumgartner 2004), but due to data limitations, the question of who leads whom has previously been unanswered (Burstein 2003). In this paper, we have contributed to answering this open question by characterizing the agenda of members of Congress and their constituents using latent topic modeling applied to the text of the tweets they sent between January 2013 and December 2014 (113th Congress). In doing so, we have been able to create fine-grained political and public agenda measures and to study not only the extent to which members of Congress follow shifts in issue attention by their constituents when deciding what issues to discuss, but also to adjudicate between three competing models of political responsiveness: whether public representatives follow changes in attention by their party supporters, the attentive public, or the general public.

We modeled how public and political agendas predict each other using a VAR model accounting for endogenous and media effects. First, we found a political responsiveness dynamic to be in place during the period of analysis. The public was not only able to lead the expressed agenda of members of Congress, but the magnitude of this phenomenon was greater than that associated with politicians’ ability to lead public agendas: an attention shift from party supporters preceded a larger shift by members of Congress than the attention shift from party supporters following an attention shift by members of Congress. Moreover, we found stronger support for some responsiveness models than others. Our findings suggest that members of Congress are mainly responsive to changes in attention allocation by party supporters and, to a lesser extent, attentive publics. The findings also suggest that mainstream media promote similar dynamics: they are particularly likely to follow the issue preferences of party supporters, and they are likely to lead the issue agenda of members of Congress. In addition, we observed Democrats and Republicans in Congress to particularly follow party supporters on issues that are relevant to them. Finally, we found very little empirical support for the claim that politicians are responsive to the general public.

These issue responsiveness findings align with the literature on policy and policy preference responsiveness that shows that political and policy agendas in the United States are mainly driven by the priorities of strong partisans (Clinton 2006; Kastellec et al. 2015; Shapiro et al. 1990). The study also supports the claim that, due to existing representation and responsiveness dynamics, political agendas are more polarized than is the American public (Grimmer 2013). While others show that this is in part a function of geographic sorting and an increasing number of clearly partisan districts (Bishop and Cushing 2008; Grimmer 2013), we show that low political attention by the general public and a higher media coverage of partisan issue preferences is also in part to blame. This has important normative implications for democratic politics, as it could be an indirect factor contributing to political polarization.

Our analysis is limited to the 113th Congress, but we argue that our findings are likely to be generalizable to the current context. Social media usage by members of Congress was already almost universal in 2013. In addition, data from the Pew Research Center show that Twitter penetration among online US adults has remained around 20% for the last five years (Smith and Anderson 2018). The fact that Twitter has become more central in US politics (Gainous and Wagner 2013), particularly after Trump was elected President, means that we would expect our findings to be more valid today and lead to more precise estimates. However, one result that may not hold today may be the notable partisan asymmetry we observe regarding the relationship between legislators and the media, which appears to be stronger for Republicans than for Democrats. This pattern could be a function of the political context, and in particular of which party is in the opposition, or it could be related to structural factors, such as the asymmetric fragmentation of the media system —currently larger on the left than on the right in the United States.

Overall, we illustrated how researchers can use social media communications to uncover agenda setting and responsiveness dynamics. Due to space constraints we had to limit the scope of our analysis, but other basic questions can be examined using this method. For example, is the President able to set political and public agendas? Previous research shows that the President’s ability is limited (Edwards and Wood 1999), but more recent studies argue that this pattern may have changed in the last few years (Lawrence and Boydstun 2017; Wells et al. 2016)—a finding that may be worth revisiting in the era of Trump’s presidency. Do politicians running in safe versus marginal districts respond to different types of constituents? Do politicians respond differently to constituents’ issue priorities depending on the issues they own? And how would these results differ across institutional or political contexts? In particular, we might have reason to expect higher levels of responsiveness in countries with higher levels of political contestation (Hobolt and Klemmensen 2008).

Another accessible topic of study is issue responsiveness at the state level. Existing responsiveness research in the United States studies how the issues discussed by Federal political elites are shaped by public issue and policy preferences (Erikson, Mackuen, and Stimson 2002; Jones and Baumgartner 2004; Page and Shapiro 1983; Stimson, Mackuen, and Erikson 1995).A relevant number of political decisions, however, are made at the state level. Are state policy makers responsive to their constituents? What type of constituents? Do federal agendas influence political discussions at the state level? Do we see differential responsiveness dynamics across states? And if so, why? In addition, given a longer time period, one could combine the fine-grained temporal measure that Twitter data offer with the curated topics of the Policy Agenda Project to determine who leads and who follows on each of the 19 issues the project has defined (Jones and Baumgartner 2004). Our hope is that both the findings and methods introduced here can serve as a springboard for research into these and other important topics related to political representation in the future.

## Footnotes

1 To be clear, we are not, however, addressing the downstream question of whether policy *outcomes* reflect the preferences of the public or particular groups of the public; see Gilens (2012) and Gilens and Page (2014) for an analysis of the determinants of policy outcomes.

2 For example, an increase in public attention to the Dakota Access Pipeline followed by Congressional hearings on a fracking bill (e.g., S.785 of the 114th Congress) would be miscategorized as a case of agenda responsiveness by a commonly used issue classification in responsiveness research, the Policy Agendas Project issue classification (Jones and Baumgartner 2004): both actions would be categorized into Natural Gas & Oil (803) within the Energy category. Although both energy related, these two issues are distinct, and assuming that Congress is reacting and being responsive to a preceding public attention change can be misleading.

3 We further test this assumption in Online Appendix A.

4 There is a fourth main theoretical model that for data limitations we are unable to test in this paper: the argument that policy makers are responsive to wealthier constituents (Gilens 2012).

5 Delli Carpini and Keeter (1996), for example, found that only30% of Americans know the name of at least one of the two Senators representing their State.

6 The authors do find, however, that when discrepancies exist between the policy preferences of some groups, politicians do tend to be more responsive to some groups: those with a higher income, education, and political sophistication.

7 As will be explained below in the *Issue Attention Congruence* section, we measure the salience of an issue by calculating the average relative daily attention that different groups of the public paid to each political issue.

8 See Online Appendix F for information regarding the data collection process.

9 A description of the procedure we followed to build this sample can be found in Online Appendix B.

10 We conducted the data collection for the random sample (General Public group) in 2015 and were constrained by Twitter’s rate limits, which do not allow downloading all of user’s tweets. In this case, part of the tweets sent in 2013 by about 44% of the random users are not included in the sample.

11 There are two reasons for this decision. First, LDA assumes that each document is a mixture of topics, which is appropriate for our conceptualization of each day’s tweets as the political agenda that each party within each legislative chamber is trying to push for that specific day. Second, conducting an analysis at the tweet level is complex, given its very limited length. The existing literature on topic modeling of tweets has found that applications that aggregate tweets by author or day outperform those that rely on individual tweets (Hong and Davison 2010).

12 Such as the ones from the Comparative Agendas Project or Manifesto Project.

13 For a general overview of the use of text-as-data methods in political science research, see Grimmer and Stewart (2013) and Wilkerson and Casas (2017).

14 To identify the list of relevant political topics, five coders used the information contained in our topic dashboard (pablobarbera.com/congress-lda) to classify each of them into three categories: non-political topics (e.g., Valentine’s Day), political topics but not related to issues (e.g., public communication by House Republicans), and political issues (e.g., gun violence, government shutdown, obamacare). Average intercoder agreement was 83% and Cronbach’s alpha was 0.92. We chose as political issues those where the modal classification (three or more coders) agreed to classify as such.

15 In this paper, we do not control for the agenda of the President for two main reasons. First, Edwards and Wood (1999) do not find the President to have a relevant agenda-setting capacity, and second, the method we employ in this paper to measure agendas (proportion of daily tweets on a set of issues) is well suited to measure group but not individual agendas, given that single individuals do not tweet frequently enough to build unbiased measures. Needless to say, revisiting the question of agenda-setting power of the President in the era of Trump would likely be a good idea for future research.

16 Democratic and Republican members of Congress, Democratic and Republican Supporters, Attentive Public, General Public, and Media.

17 We run Augmented Dickey–Fuller unit root tests and confirm that the series are stationary.

18 Autocorrelation and partial autocorrelation functions vary depending on the group and issue series one explores. However, on average we observe autocorrelations to go below 0.1 after 5–9 days, and partial autocorrelations to be below this level after 3–5 days; which indicates that by using a seven-lag structure we are accounting and controlling for the autocorrelation nature of these variables.

19 As we point out in the *Discussion*, we believe that future work should focus on studying how the effects presented here are conditional on the issue or issue type at hand. The methods advanced here can, for example, be used to clearly test hypotheses on the relationship between issue responsiveness and issue ownership. This is not, however, the immediate purpose of this study.

20 In Online Appendix D, we provide results based on shorter and longer term IRFs.

21 When calculating the IRFs for the permanent 10-percentage-point change , we insert a new increase of attention every day to the covariate of interest until the predicted attention for that group reaches 10% without the need of any extra shock. If we let *φ* represent the attention increase we introduce in the covariate, and *ŷ* the resulting predicted value attention for that same covariate, then we can formally express *φ* during the 15 day period as follows: during the 15 day period as follows: ϕi,t=10− y^ if t is > 010, if t is 0

22 See Online Appendix E.1.

23 In Online Appendix C, we show predicted effects for all issues.

## SUPPLEMENTARY MATERIAL

To view supplementary material for this article, please visit https://doi.org/10.1017/S0003055419000352.

Replication materials can be found on Dataverse at: https://doi.org/10.7910/DVN/AA96D2.

## References

[cit0001] Adler Scott, and John Wilkerson. 2012 Congress and the Politics of Problem Solving. New York: Cambridge University Press.

[cit0002] Aldrich John H. 1995 Why Parties? The Origin and Transformation of Political Parties in America. Chicago: The University of Chicago Press.

[cit0002a] Anand N., and Peterson Richard A. 2000 “When Market Information Constitutes Fields: Sensemaking of Markets in the Commercial Music Industry.” Organization Science 11 (3): 270–84.

[cit0003] Arnold R. Douglas. 1990 The Logic of Congressional Action. New Haven: Yale University Press.

[cit0004] Baumgartner Frank R., and Jones Bryan D. 1993 Agendas and Instability in American Politics. Chicago: University of Chicago Press.

[cit0005] Bawn Kathleen, Cohen Martin, Karol David, Masket Seth, Noel Hans, and Zaller John 2012 “ATheory of Political Parties: Groups, Policy Demands and Nominations in American Politics.” Perspectives on Politics 10 (3): 571–97.

[cit0006] Birkland Thomas A. 1998 “Focusing Events, Mobilization, and Agenda Setting.” Journal of Public Policy 18 (1): 53–74.

[cit0007] Bishop Bill, and Cushing Robert G. 2008 The Big Sort: Why the Clustering of Like-Minded America Is Tearing Us Apart. NewYork: Houghton Mifflin.

[cit0008] Blei David M., Ng Andrew Y., and Jordan Michael I. 2003 “Latent Dirichlet Allocation.” The Journal of Machine Learning Research 3: 993–1022.

[cit0009] Boydstun Amber E. 2013 Making the News: Politics, the Media, and Agenda Setting. Chicago: University of Chicago Press.

[cit0010] Burstein Paul. 2003 “The Impact of Public Opinion on Public Policy: A Review and an Agenda.” Political Research Quarterly 56 (1): 29–40.

[cit0011] Burstein Paul. 2014 American Public Opinion, Advocacy, and Policy in Congress: What the Public Wants and what it Gets. New York: Cambridge University Press.

[cit0012] Campbell Angus, Converse Philip E., Miller Warren E., and Stokes Donald E. 1960 The American Voter. New York: John Wiley.

[cit0013] Campbell James E., Dettrey Bryan J., and Yin Hongxing 2010 “The Theory of Conditional Retrospective Voting: Does the Presidential Record Matter Less in Open-Seat Elections?” The Journal of Politics 72 (4): 1083–95.

[cit0014] Canes-Wrone Brandice, and Shotts Kenneth W. 2004 “The Conditional Nature of Presidential Responsiveness to Public Opinion.” American Journal of Political Science 48 (4): 690–706.

[cit0015] Andreu Casas, and Morar David 2015 “Different Channel, Same Strategy? Filling Empirical Gaps in congress Literature.” In Proceedings of the Annual Meeting of the American Political Science Association, eds. Mosley Layna and Tillery Alvin Bernard San Francisco: American Political Science Association 1–21. Available at SSRN: https://ssrn.com/abstract=3395307.

[cit0016] Caughey Devin, and Warshaw Christopher 2018 “Policy Preferences and Policy Change: Dynamic Responsiveness in the American States, 1936–2014.” American Political Science Review 112 (2): 249–66.

[cit0017] Chang Jonathan, Gerrish Sean, Wang Chong, Boyd-graber Jordan L., and Blei David M. 2009 “Reading Tea Leaves: How Humans Interpret Topic Models.” In Advances in Neural Information Processing Systems. Vol. 22, eds.: Bengio Yoshua, Schuurmans Dale, Lafferty John, Williams Chris, and Culotta Aaron Vancouver, BC: Curran Associates, Inc., 288–96.

[cit0018] Chen Kaiping, Lee Nathan, and Marble William 2018 “Do Elected Officials Listen to Constituents on Social Media? Survey Evidence from Local Politicians in the United States.” Working paper. Available at SSRN: https://ssrn.com/abstract=3251651 or http://dx.doi.org/10.2139/ssrn.3251651.

[cit0019] Feng Chi, and Yang Nathan 2011 “Twitter Adoption in congress.” Review of Network Economics 10 (1): 1–46.

[cit0020] Clinton Joshua D. 2006 “Representation in congress: Constituents and Roll Calls in the 106th House.” The Journal of Politics 68 (2): 397–409.

[cit0021] Converse Philip E. 2006 “The Nature of Belief Systems in Mass Publics.” Critical Review 18 (1–3): 1–74.

[cit0022] Delli Carpini Michael X., and Keeter Scott 1996 What Americans Know about Politics and Why it Matters. New Haven: Yale University Press.

[cit0023] de Vreese Claes H., and Boomgaarden Hajo 2006 “News, Political Knowledge and Participation: The Differential Effects of News media Exposure on Political Knowledge and Participation.” Acta Politica 41 (4): 317–41.

[cit0024] Downs Anthony. 1957 An Economic Theory of Democracy. New York: Harper and Row.

[cit0025] Edwards George C., and Wood B. Dan 1999 “Who Influences Whom? The President, congress, and the media.” American Political Science Review 93 (2): 327–44.

[cit0026] Egan Patrick J. 2013 Partisan Priorities: How Issue Ownership Drives and Distorts American Politics. New York: Cambridge University Press.

[cit0027] Enders Walter, and Sandler Todd 1993 “The Effectiveness of Antiterrorism Policies: A Vector-Autoregression-Intervention Analysis.” American Political Science Review 87 (4): 829–44.

[cit0028] Erikson Robert S., Mackuen Michael B., and Stimson James A. 2002 The Macro Polity. New York: Cambridge University Press.

[cit0029] Evans Heather K., Cordova Victoria, and Sipole Savannah 2014 “Twitter Style: An Analysis of How House Candidates Used Twitter in Their 2012Campaigns.” PS: Political Science&Politics 47 (2): 454–62.

[cit0030] Feezell Jessica T. 2018 “Agenda Setting through Social media: The Importance of Incidental News Exposure and Social Filtering in the Digital Era.” Political Research Quarterly 71 (2): 482–94.

[cit0031] Fenno Richard F. 1978 Home Style: House Members in Their Districts. London: Longman.

[cit0032] Freeman John R., Williams John T., and Lin Tse-min 1989 “Vector Autoregression and the Study of Politics.” American Journal of Political Science 33 (4): 842–77.

[cit0033] Gainous Jason, and Wagner Kevin M. 2013 Tweeting to Power: The Social Media Revolution in American Politics. New York: Oxford University Press.

[cit0034] Geer J.G. 1996 From Tea Leaves to Opinion Polls: A Theory of Democratic Leadership. New York: Columbia University Press.

[cit0035] Gerber Alan S., Karlan Dean, and Bergan Daniel 2009 “Does the media Matter? A Field experiment Measuring the Effect of Newspaperson Voting Behavior and Political Opinions.” American Economic Journal: Applied Economics 1 (2): 35–52.

[cit0036] Gerber Elisabeth R., and Morton Rebecca B. 1998 “Primary Election Systems and Representation.” Journal of Law, Economics, and Organization 14 (2): 304–24.

[cit0037] Gilens Martin. 2012 Affluence and Influence: Economic Inequality and Political Power in America. Princeton, New Jersey: Princeton University Press.

[cit0038] Gilens Martin, and Page Benjamin I. 2014 “Testing Theories of American Politics: Elites, Interest Groups, and Average Citizens.” Perspectives on Politics 12 (3): 564–81.

[cit0039] Gitlin Todd. 1980 The Whole World Is Watching: Mass Media in the Making & Unmaking of the New Left. Berkeley, CA: University of California Press.

[cit0040] Golbeck Jennifer, Grimes Justin M., and Rogers Anthony 2010 “Twitter Use by the US Congress.” Journal of the American Society for Information Science and Technology 61 (8): 1612–21.

[cit0041] Graber Doris A. 1997 Mass Media and American Politics. Washington, D.C: CQ Press.

[cit0042] Grimmer Justin. 2013 “Appropriators Not Position Takers. the Distorting Effects of Electoral Incentives on Congressional Representation.” American Journal of Political Science. 57 (3): 624–42.

[cit0043] Grimmer Justin, and Stewart Brandon M. 2013 “Text as Data: The Promise and Pitfalls of Automatic Content Analysis Methods for Political Texts.” Political Analysis 21 (3): 267–97.

[cit0044] Habel Philip D. 2012 “Following the Opinion Leaders? The Dynamics of Influence Among media Opinion, the Public, and Politicians.” Political Communication 29 (3): 257–77.

[cit0045] Hemphill Libby, Otterbacher Jahna, and Shapiro Matthew 2013 “What’s Congress Doing on Twitter?” In Proceedings of the 2013 Conference on Computer Supported Cooperative Work, eds. Bruckman Amy, Counts Scott, Lampe Cliff, and Terveen Loren New York: ACM, 877–86.

[cit0046] Hemphill Libby, and Roback Andrew J. 2014 “Tweet Acts: How Constituents Lobby congress via Twitter.” In Proceedings of the 17th ACM Conference on Computer Supported Cooperative Work & Social Computing, eds. Fussell Susan, Lutters Wayne, Morris Meredith Ringel, and Reddy Madhu New York: ACM, 1200–10.

[cit0047] Hibbing John R., and Theiss-Morse Elizabeth 2002 Stealth Democracy: Americans’ Beliefs about How Government Should Work. Cambridge, UK: Cambridge University Press.

[cit0048] Hobolt Sara, and Klemmensen Robert 2008 “Government Responsiveness and Political Competition in Comparative Perspective.” Comparative Political Studies 41 (3): 309–37.

[cit0049] Holbrook Thomas M., and McClurg Scott D. 2005 “The Mobilization of Core Supporters: Campaigns, Turnout, and Electoral Composition in United States Presidential Elections.” American Journal of Political Science 49 (4): 689–703.

[cit0050] Hong Liangjie, and Davison Brian D. 2010 “Empirical Study of Topic Modeling in Twitter.” In Proceedings of the First Workshop on Social Media Analytics, eds. Melville Prem, Leskovec Jure, and Provost Foster New York: ACM, 80–8.

[cit0051] Iyengar Shanto, and Kinder Donald R. 2010 News that Matters: Television and American Opinion. Chicago: University of Chicago Press.

[cit0052] Jacobs Lawrence R., and Shapiro Robert Y. 1997 “The Myth of the Pandering Politician.” The Public Perspective 8: 3–5.

[cit0053] Jacobs Lawrence R., and Shapiro Robert Y. 2000 Politicians Don’t Pander: Political Manipulation and the Loss of Democratic Responsiveness*:* University of Chicago Press.

[cit0054] Jones Bryan D. 2004 Reconceiving Decision-Making in Democratic Politics: Attention, Choice, and Public Policy. Chicago: The University of Chicago Press.

[cit0055] Jones Bryan D., and Baumgartner Frank R. 2004 “Representation and Agenda Setting.” Policy Studies Journal 32 (1): 1–24.

[cit0056] Jones Bryan D., and Baumgartner Frank R. 2005 The Politics of Attention: How Government Prioritizes Problems. Chicago: University of Chicago Press.

[cit0057] Kastellec Jonathan P., Lax Jeffrey R., Malecki Michael, and Phillips Justin H. 2015 “Polarizing the Electoral Connection: Partisan Representation in Supreme Court Confirmation Politics.” The Journal of Politics 77 (3): 787–804.

[cit0058] Katz Elihu, and Lazarsfeld Paul F. 1955 Personal Influence: The Party Played by People in the Flow of Mass Communications. New York: Free Press.

[cit0059] King Gary, Schneer Benjamin, and White Ariel 2017 “How the News media Activate Public Expression and Influence National Agendas.” Science 358 (6364): 776–80. 29123065 10.1126/science.aao1100

[cit0060] Kingdon John W. 2013 Agendas, Alternatives, and Public Policies. London, UK: Pearson Education Limited.

[cit0061] Ladd Jonathan M., and Lenz Gabriel S. 2009 “Exploiting a Rare Communication Shift to Document the Persuasive Power of the Newsmedia.” American Journal of Political Science 53 (2): 394–410.

[cit0062] Lau Richard R., and Redlawsk David P. 2006 How Voters Decide: Information Processing in Election Campaigns. New York: Cambridge University Press.

[cit0063] Lawrence Regina G., and Boydstun Amber E. 2017 “What We Should Really Be Asking about media Attention to Trump.” Political Communication 34 (1): 150–3.

[cit0064] Lax Jeffrey R., and Phillips Justin H. 2011 “The Democratic Deficit in the States.” American Journal of Political Science 56 (1): 148–66.

[cit0065] Lupia Arthur, McCubbins Mathew D., and Arthur Lupia 1998 The Democratic Dilemma: Can Citizens Learn what They Need to Know? New York: Cambridge University Press.

[cit0066] McCombs Maxwell E., and Shaw Donald L. 1972 The Agenda-Setting Function of Mass media. Public Opinion Quarterly 36 (2): 176–87.

[cit0067] Neundorf Anja, and Adams James 2018 “The Micro-foundations of Party Competition and Issue Ownership: The Reciprocal Effects of Citizens’ Issue Salience and Party Attachments.” British Journal of Political Science 48 (2): 385–406.

[cit0068] O’Connor Brendan, Balasubramanyan Ramnath, Routledge Bryan R., and Smith Noah A. 2010 “From Tweets to Polls: Linking Text Sentiment to Public Opinion Time Series.” In Fourth International AAAI Conference on Weblogs and Social Media, eds. Hears Marti, Cohen William, and Gosling Samuel Menlo Park, CA: The AAAI Press, 122–8.

[cit0069] Page Benjamin I. 1994 “Democratic Responsiveness? Untangling the Links between Public Opinion and Policy.” PS: Political Science and Politics 27 (1): 25–9.

[cit0070] Page Benjamin I., and Shapiro Robert Y. 1983 “Effects of Public Opinion on Policy.” American Political Science Review 77 (1): 175–90.

[cit0071] Page Benjamin I., and Shapiro Robert Y. 1992 The Rational Public: Fifty Years of Trends in American’s Policy Preferences. Chicago: The University of Chicago Press.

[cit0072] Quinn Kevin M., Monroe Burt L., Colaresi Michael, Crespin Michael H., and Radev Dragomir R. 2010 “How to Analyze Political Attention with Minimal Assumptions and Costs.” American Journal of Political Science 54 (1): 209–28.

[cit0073] Russell Annelise. 2018a “The Politics of Prioritization: Senators’ Attention in 140 Characters.” The Forum 16 (2): 331–56.

[cit0074] Russell Annelise. 2018b “USSenators on Twitter: Asymmetric Party Rhetoric in 140 Characters.” American Politics Research 46 (4): 695–723.

[cit0075] Schattschneider Elmer E. 1975 The Semisovereign People: A Realist’s View of Democracy in America. New York: Dryden Press.

[cit0076] Schlozman Kay Lehman, Verba Sidney, and Brady Henry E. 2012 The Unheavenly Chorus: Unequal Political Voice and the Broken Promise of American Democracy. Princeton, NJ: Princeton University Press.

[cit0077] Shapiro Catherine R. Brody David W. Brady Richard A. , and Ferejohn John A. 1990 “Linking Constituency Opinion and Senate Voting Scores: A Hybrid Explanation.” Legislative Studies Quarterly 15 (4): 599–621.

[cit0078] Shapiro Matthew A. , and Hemphill Libby 2017 “Politicians and the Policy Agenda: Does Use of Twitter by the US Congress Direct New York Times Content?” Policy & Internet 9 (1): 109–32.

[cit0079] Shapiro Matthew A. Hemphill Libby and Otterbacher Jahna 2012 “Doing what I Say: Connecting Congressional Social media Behavior and Congressional Voting.” In Proceedings of the Annual Meeting of the Midwest Political Science Association, eds. Rogowski Jon, Sinclair Betsy Chicago: Midwest Political Science Association, 1–32.

[cit0080] Shoemaker Stephen, and Reese Pamela 1996 Mediating the Message: Theories of Influences on Mass media Content. New York: Longman.

[cit0081] Sims Christopher A. 1980 “Macroeconomics and Reality.” Econometrica 48 (1): 1–48.

[cit0082] Smith Aaron, and Anderson Monica 2018 Social Media Use in 2018. Washington, DC: Pew Internet&American Life Project Retrieved March 3, 2018.

[cit0083] Sniderman Paul M. Brody Richard A. and Tetlock Phillip E. 1993 Reasoning and Choice: Explorations in Political Psychology. New York: Cambridge University Press.

[cit0084] Soroka Stuart N. 2002 “Issue Attributes and Agenda-Setting by media, the Public, and Policymakers in Canada.” International Journal of Public Opinion Research 14 (3): 264–85.

[cit0085] Soroka Stuart N. , and Wlezien Christopher 2009 Degrees of Democracy: Politics, Public Opinion, and Policy. New York: Cambridge University Press.

[cit0086] Stimson James A. Mackuen Michael B. , and Erikson Robert S. 1995 “Dynamic Representation.” American Political Science Review 89 (3): 543–65.

[cit0087] Sulkin Tracy. 2005 Issue Politics in Congress. New York: Cambridge University Press.

[cit0088] Tausanovitch Chris, and Warshaw Christopher 2014 “Representation in Municipal Government.” American Political Science Review 108 (3): 605–41.

[cit0089] Walgrave Stefaan, Soroka Stuart, and Nuytemans Michiel 2008 “The Mass media’s Political Agenda-Setting Power: ALongitudinal Analysis of Media, Parliament, and Government in Belgium (1993 to 2000).” Comparative Political Studies 41 (6): 814–36.

[cit0090] Walgrave Stefaan, and Aelst Peter Van 2006 “The Contingency of the Mass Media’s Political Agenda Setting Power: Toward a Preliminary Theory.” Journal of Communication 56 (1): 88–109.

[cit0091] Wallis KennethF. 1987 “Time Series Analysis of Bounded Economic Variables.” Journal of Time Series Analysis 8 (1): 115–23.

[cit0092] Webster James G. , and Ksiazek Thomas B. 2012 “The Dynamics of Audience Fragmentation: Public Attention in an Age of Digital media.” Journal of Communication 62 (1): 39–56.

[cit0093] Wells Chris, Shah Dhavan V., Pevehouse Jon C., Yang JungHwan, Pelled Ayellet, Boehm Frederick, Lukito Josephine, Ghosh Shreenita,and Schmidt Jessica L. 2016 “How Trump Drove Coverage to the Nomination: Hybrid Media Campaigning.” Political Communication 33 (4): 669–76.

[cit0094] Wilkerson John, and Casas Andreu 2017 “Large-scale Computerized Text Analysis in Political Science: Opportunities and Challenges.” Annual Review of Political Science 20 (1): 529–44.

[cit0095] Wlezien Christopher. 2004 “Patterns of Representation: Dynamics of Public Preferences and Policy.” The Journal of Politics 66 (1): 1–24.

[cit0096] Wood B. Dan, and Peake Jeffrey S. 1998 “The Dynamics of Foreign Policy Agenda Setting.” American Political Science Review 92 (1): 173–84.

[cit0097] Wouters Ruud, and Walgrave Stefaan 2017 “Demonstrating Power: How Protest Persuades Political Representatives.” American Sociological Review 82 (2): 361–83.

[cit0098] Wright Gerald C. 1989 “Policy Voting in the US Senate: Who Is Represented?” Legislative Studies Quarterly 14 (4): 465–86.

[cit0099] Zaller John R. 1992 The Nature and Origins of Mass Opinion. New York: Cambridge University Press.

