# Supplementary material for: Who Leads? Who Follows? Measuring Issue Attention and Agenda Setting by Legislators and the Mass Public Using Social Media Data
Source: Am Polit Sci Rev. 2019 Jul 12;113(4):883–901. doi: 10.1017/S0003055419000352 (PMC7672368; doi:10.1017/S0003055419000352)
Supplement: Supplementary file 1 [file APSR-113-04-883-s001.pdf]

SUPPLEMENTARY MATERIALS: Who Leads? Who Follows? Measuring Issue Attention and Agenda Setting by Legislators and the Mass Public Using Social Media Data

CONTENTS

A Validation of Public Agenda Measures 2

B Procedure to Elaborate the Random Sample of U.S. Twitter Users 5

C Complete Issue-Level Results 6

D Full 60-day Impulse Response Functions 9

E Robustness Checks 11

E.1 Exploring Election-Year Effects: Modeling 2013 and 2014 Data Separately 11

E.2 Modeling Broader Political Issues 12

E.3 Modeling an Existing Classification of Narrow Political Issues 14

E.4 Modeling Topics Discovered in All Tweets 15

E.5 Rulling out a Media-Collider Bias 18

F Additional descriptive statistics 24

F.1 Members of Congress on Twitter 24

F.2 Party Supporters on Twitter 25

G Topic Modeling of Tweets by Legislators and Citizens 26

G.1 Overview of Latent Dirichlet Allocation Model 26

G.2 Choosing the Number of Topics of the LDA Model 26

G.3 Validation of Discovered Topics 28

G.4 Attention to political issues by legislators and citizens 29

References 32

A. VALIDATION OF PUBLIC AGENDA MEASURES

Research studying the correspondence between the issues politicians and the public discuss has traditionally used Gallup's *Most Important Problem* (MIP) polls to measure the issue priorities of the public – see for example Jones and Baumgartner (2004). For decades, Gallup has been asking the same (or very similar) question to the American public "What is the most important problem facing the nation today?" Some have argued that using Gallup's MIP as a measure of the public agenda is problematic because the wording of the question has slightly changed over time (Soroka, 2002, "Number of Responses and the Most Important Problem") and because it is unclear whether it is measuring issue salience or problem perception (Wlezien, 2005, *Electoral Studies*). Others have argued however that, despite its pitfalls, Gallup's MIP is the best data source available to measure what issues are salient to the public (Jones and Baumgartner 2004).

In the paper we pointed out an additional downside related to Gallup's MIP polls: they aggregate monthly issue attention, which does not facilitate uncovering whether elite political agendas influence public attention, or the other way around, if such influence is happening more quickly than one would observe with monthly data. We also argued that public attention measures created using Twitter data provide more detailed information and facilitate studying temporal patterns. Nevertheless, analyses based on tweets about politics are subject to potential biases: not all citizens have a Twitter account, nor do all those who do tweet often. In this appendix we perform some construct validity tests and asses the extent to which our Twitter-constructed public agendas are a valid measure of the issues different groups of the public pay attention to. To do so, for the period of analysis we correlate monthly MIP responses and our Twitter-constructed public agendas. We expect a positive correlation between the two, but given that MIP polls not only capture salience but also longer-term issue priorities (Wlezien, 2005, *Electoral Studies*), we expect such correlation not to be perfect.

We collect Gallup's MIP data from January 2013 though December 2014 from the Roper Center.[1] The data contains individual MIP responses that have been manually coded according to the 19 issue-classification of the Comparative Agendas Project (CAP).[2] These are responses to monthly polls, but as there are a few scattered months for which no data is available, we aggregate these individual responses on a quarterly basis: calculating the proportion of all responses in each three-month period that are about each of the 19 CAP issue categories. We also aggregate the responses by different groups of individuals based on party identification: Democrats, Weak Democrats, Independents, Republicans, and Weak Republicans).

Then we assign one of the 19 CAP issue categories to each of our political issues uncovered from the topic modelling described above. Table A1 shows the CAP codes assigned to our 46 political issues. Then, for each group of the public in our analysis (Democratic and Republican supporters, the attentive public, and the general public), we also aggregate in a quarterly basis the estimated Twitter attention to

[1]The data is available from the following link.

[2]The codebook for the Comparative Agendas Project issue-classification is available using the following link.

APSR Submission Template APSR Submission Template APSR Submission Template APSR Submission Template APSR Submission Template APSR Submission Template APSR Submission Template APSR Submission Template

each of the CAP issues. At this point, both measures (the MIP and Twitter-based measures) are in the same unit of analysis (quarterly attention to the 19 CAP issues) and ready to be compared.

Table A2 shows Pearson correlations between these MIP and Twitter-based public agenda measures. All correlations are positive and most of them are of substantive magnitude. We see a very strong correlation between the Twitter-based measure of the agenda of Democratic and Republican supporters and the issues all poll respondents indicated as the most important (.46 and .69 correlation, respectively). If we break down these correlation by party identification, we see how our measure of the agenda of Democratic supporters is more strongly correlated with MIP responses by Democrats (.49) than by Republicans (.41). And we observe the same pattern for Republican supporters. Our measure of their agenda is more strongly correlated with MIP responses by Republicans (.70) than by Democrats (.68). Moreover, although of a slightly smaller magnitude, we also observe substantive positive correlations between our Twitter-based measures of the agenda of the attentive and the general public, and Gallup's MIP responses: Pearson correlations of between .32 and .4.

TABLE A2. Pearson correlation between Twitter-based Public Agenda Measures and Gallup's MIP polls

	Gallup MIP Responses					
**Twitter Measure**	Full Sample	Democrat	Weak Democrat	Independent	Weak Republican	Republican
Democratic Supporters	0.46	0.49	0.46	0.43	0.41	0.45
Republican Supporters	0.69	0.68	0.68	0.66	0.67	0.70
Attentive Public	0.35	0.35	0.34	0.35	0.33	0.35
General Public	0.37	0.39	0.37	0.38	0.32	0.35

APSR Submission Template

TABLE A1. Comparative Agendas Project codes assigned to our political issues

Topic #	Our Label	CAP Major	CAP Minor
3	Investigation of Benghazi Attack	16: Defense	1619: Foreign Operations
7	100 Days of #BringBackOurGirls campaign	19: International Affairs	1927: Terrorism
9	Gender Wage Gap	2: Civil Rights	202: Gender Discrimination
11	Hobby Lobby SC Decision (Dem.)	2: Civil Rights	207: Freedom of Speech
12	Republican Issues Spring 2013	6: Education	600: General
14	Marriage Equality	2: Civil Rights	202: Gender Discrimination
15	Gun Violence	12: Law and Crime	1299: Other
16	Abortion (Pro-Life)	2: Civil Rights	208: Right to Privacy
17	2013 Government Shutdown (Rep.)	1: Macroeconomics	105: National Budget
18	Veteran Affairs Delays Scandal	16: Defense	1608: Personnel Issues
20	NSA Surveillance Scandal	16: Defense	1603: Intelligence
23	#BringBackOurGirls campaign	19: International Affairs	1927: Terrorism
26	2013 Government Shutdown (Democrats)	1: Macroeconomics	105: National Budget
27	Student Debt (2014)	6: Education	601: Higher Education
28	Employment Non-Discrimination Act	5: Labor	505: Fair Labor Standards
32	Islamic State	16: Defense	1619: Foreign Operations
33	Use of Military Force in Syria	16: Defense	1619: Foreign Operations
35	2013 Budget Sequestration (Republicans)	1: Macroeconomics	105: National Budget
36	Ebola	3: Health	331: Disease Prevention
37	Social Security	13: Social Welfare	1300: General
38	Budget Discussion (early 2014)	1: Macroeconomics	105: National Budget
39	Keystone XL Pipeline	8: Energy	803: Natural Gas and Oil
41	Immigration (Border Security)	9: Immigration	900: General
42	2013 Budget Sequestration (Democrats)	1: Macroeconomics	105: National Budget
43	Executive Action on Immigration	9: Immigration	900: General
46	Unemployment Numbers Reports	1: Macroeconomics	103: Unemployment Rate
47	Paul Ryan Budget Proposal	1: Macroeconomics	105: National Budget
48	Black History Month	2: Civil Rights	201: Minority Discrimination
49	2013 Budget Agreement	1: Macroeconomics	105: National Budget
50	Climate Change	7: Environment	705: Air Pollution
51	Lame Duck Congress	20: Government Operations	2099: Other
53	Minimum Wage	5: Labor	505: Fair Labor Standards
56	Student Debt (2013)	6: Education	601: Higher Education
58	Affordable Care Act	3: Health	301: Health Care Reform
59	Budget Discussion (mid-2014)	1: Macroeconomics	105: National Budget
62	Border Crisis in Texas	9: Immigration	900: General
63	Obamacare (Employer Mandate)	3: Health	301: Health Care Reform
64	FAA Furloughs Cause Flight Delays	20: Government Operations	2099: Other
66	Malaysian Airlines Crash in Ukraine	19: International Affairs	1921: Specific Country
67	Comprehensive Immigration Reform	9: Immigration	900: Immigration
70	#MiddleClassFirst campaign	1: Macroeconomics	107: Tax Code
74	Hobby Lobby SC Decision (Rep.)	2: Civil Rights	207: Freedom of Speech
75	Military Justice Improvement Act	16: Defense	1608: Personnel Issues
81	Poverty (SNAP program)	13: Social Welfare	1302: Low-Income Assistance
83	21st Century Cures Initiative	3: Health	398: R&D
85	Unemployment Insurance	5: Labor	503: Employee Benefits
88	IRS Scandal	1: Macroeconomics	107: Tax Code
89	Obamacare (Website and Implementation)	3: Health	301: Health Care Reform
93	Jobs Bills Omnibus	5: Labor	500: General
96	Violence Against Women Act	2: Civil Rights	202: Gender Discrimination
97	Protests in Ukraine and Venezuela	19: International Affairs	1921: Specific Country
99	CIA Detentions and Interrogations Report	16: Defense	1603: Intelligence
100	#ObamacareInThreeWords Campaign	3: Health	301: Health Care Reform
101	Student Debt	6: Education	601: Higher Education
102	Hobby Lobby SC Decision	2: Civil Rights	207: Freedom of Speech
103	Budget Discussion	1: Macroeconomics	105: National Budget
104	2013 Government Shutdown	1: Macroeconomics	105: National Budget

APSR Submission Template APSR Submission Template APSR Submission Template APSR Submission Template APSR Submission Template APSR Submission Template APSR Submission Template APSR Submission Template

B. PROCEDURE TO ELABORATE THE RANDOM SAMPLE OF U.S. TWITTER USERS

FIGURE A1. Flowchart describing the method used to elaborate the random sample of 25,000 U.S. Twitter users

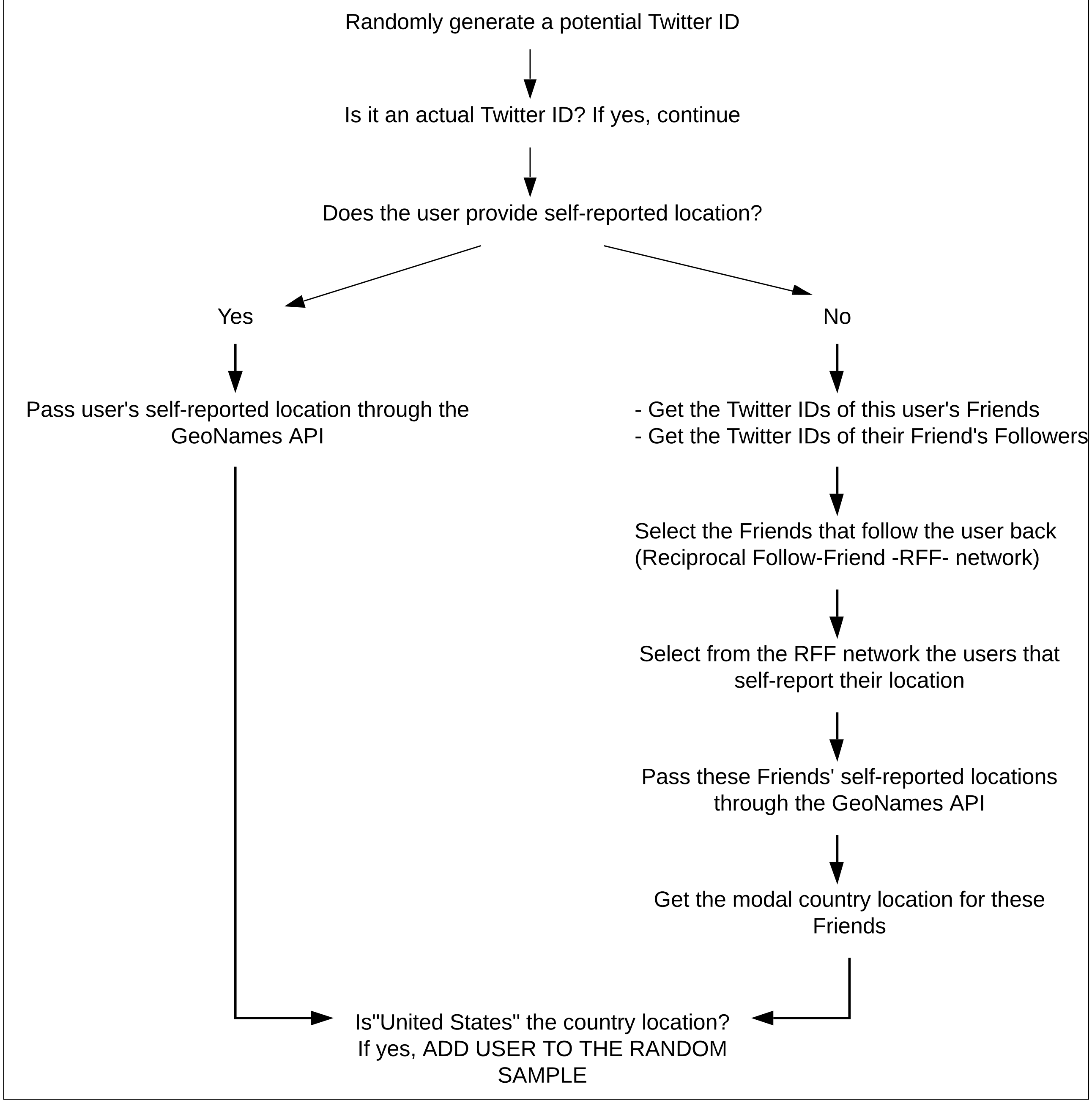

C. COMPLETE ISSUE-LEVEL RESULTS

In Figure 4 we reported issue-level responsiveness results. To avoid overcrowding the figure, we only reported coefficients that did not cross zero. In here we include two new versions of the same figure. Figure A2 reports all the coefficients and Figure A3 shows only those that do cross zero (so the ones not included in Figure 4). Finally, in TableA3 we provide a count of the issues for which a shift in an attention by a given group had a statistically significant effect on another group.

FIGURE A2. Predicted Issue Responsiveness Across Issues and Groups.

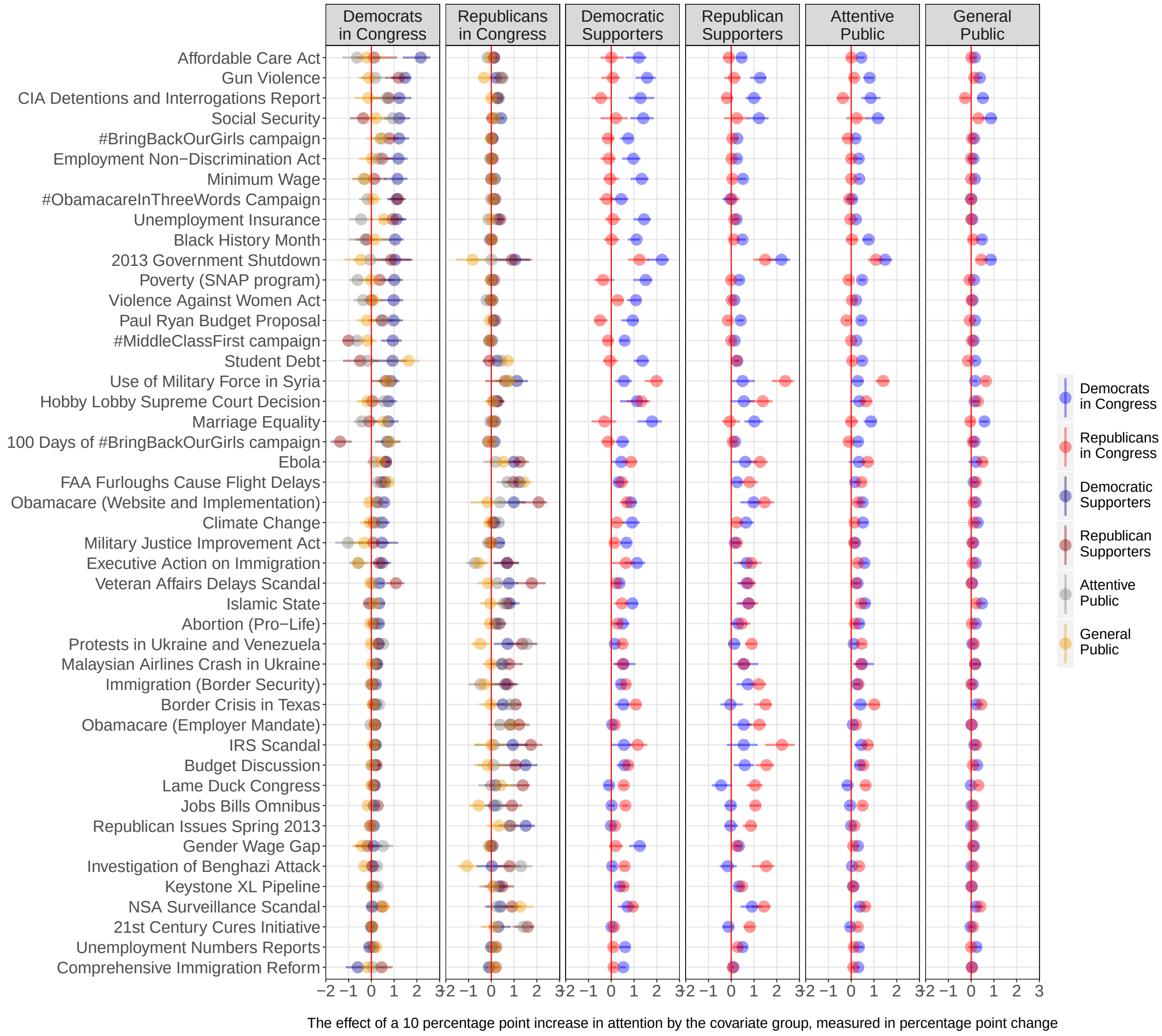

Note: The coefficients (with 95% confidence intervals) indicate (in percentage points) how much more/less cumulative attention the groups in the panel titles paid to the issue in the y-axis as a result of a group (identified by the color) increasing the attention to the same issue by 10 percentage points 15 days ago. All coefficients have been included. The two left-most panels show the influence of the public on Members of Congress. The four right-most panels show the influence of Democratic and Republican members of Congress on the public.

APSR Submission Template

APSR Submission Template

FIGURE A3. Predicted Issue Responsiveness Across Issues and Groups.

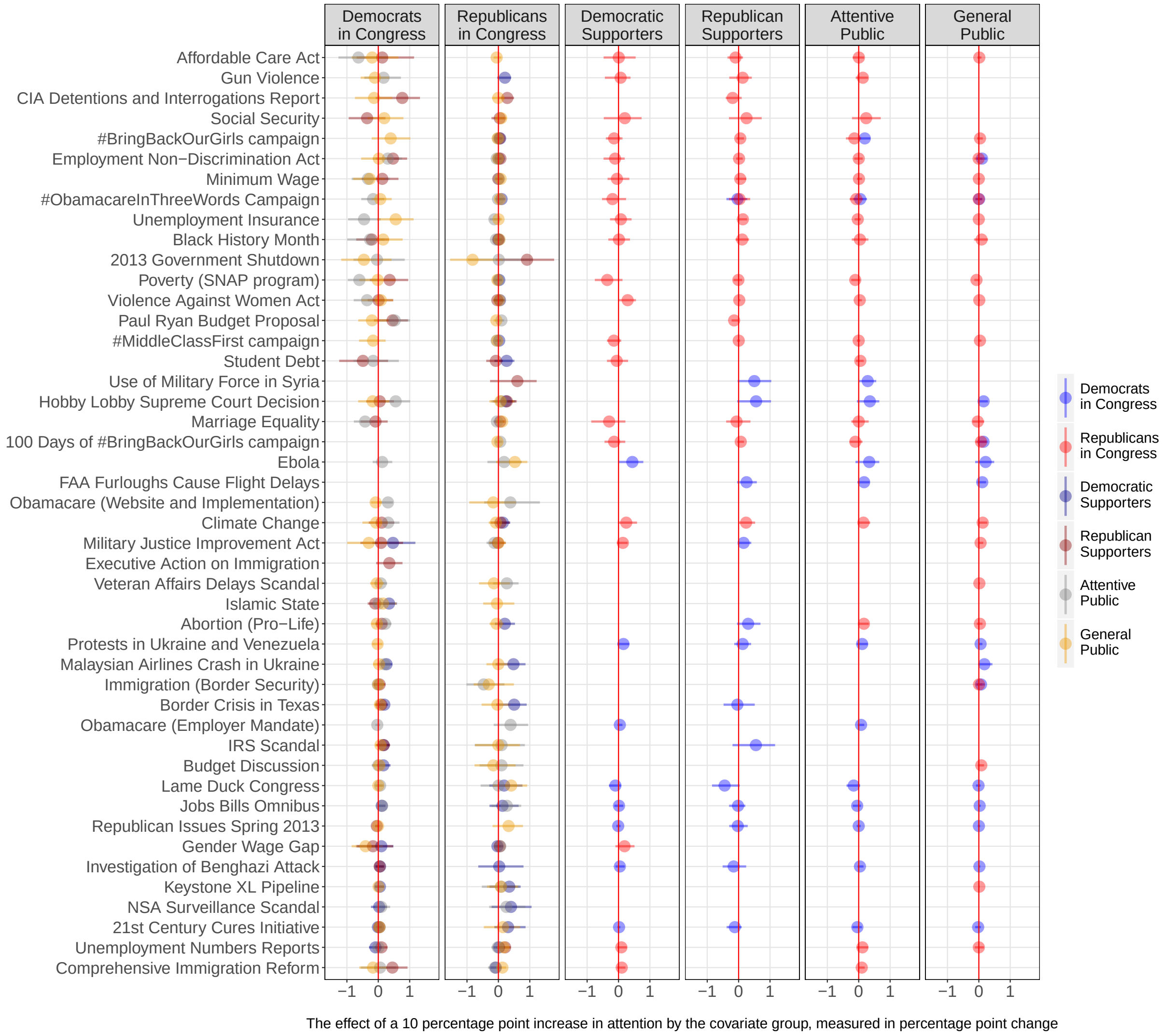

Note: The coefficients (with 95% confidence intervals) indicate (in percentage points) how much more/less cumulative attention the groups in the panel titles paid to the issue in the y-axis as a result of a group (identified by the color) increasing the attention to the same issue by 10 percentage points 15 days ago. Only coefficients crossing zero have been included. The two left-most panels show the influence of the public on Members of Congress. The four right-most panels show the influence of Democratic and Republican members of Congress on the public.

TABLE A3. Number of topics for which an attention shift by a given group had a statistically significant effect on another group.

Covariate	Outcome	Num. of Significant Topic Effects
Democrats in Congress	Democratic Supporters	39/46
Democrats in Congress	Republican Supporters	33/46
Democrats in Congress	Attentive Public	34/46
Democrats in Congress	General Public	24/46
Republicans in Congress	Democratic Supporters	24/46
Republicans in Congress	Republican Supporters	28/46
Republicans in Congress	Attentive Public	26/46
Republicans in Congress	General Public	12/46
Democratic Supporters	Democrats in Congress	32/46
Democratic Supporters	Republicans in Congress	22/46
Republican Supporters	Democrats in Congress	18/46
Republican Supporters	Republicans in Congress	28/46
Attentive Public	Democrats in Congress	16/46
Attentive Public	Republicans in Congress	19/46
General Public	Democrats in Congress	4/46
General Public	Republicans in Congress	4/46

APSR Submission Template APSR Submission Template APSR Submission Template APSR Submission Template APSR Submission Template APSR Submission Template APSR Submission Template APSR Submission Template

APSR Submission Template APSR Submission Template APSR Submission Template APSR Submission Template APSR Submission Template APSR Submission Template APSR Submission Template APSR Submission Template

D. FULL 60-DAY IMPULSE RESPONSE FUNCTIONS

In the paper we use 15-day Cummulative Impulse Response Functions (IRFs) to study responsiveness dynamics among the different groups of analysis. We could have used shorter or longer term IRFs: shorter-term IRFs would have revealed smaller effects while longer ones would have shown stronger effects. We believe that 15 days is a reasonable time window: it allows for the seven lags included in the model to come into effect and for all the reciprocal channels of influence to be activated, while still keeping the simulated scenario in the realm of what is feasible (we can envision most political discussions to go for about a week or two, but probably not for much longer).

Nevertheless, in this appendix we provide full 60-day cumulative IRFs estimated (Figure A4) to show that the reported 15-day IRFs are indeed a middle ground between low immediate effects and a much stronger (but rather unfeasible) long-term influence.

FIGURE A4. 60 day Cumulative Impulse Response Functions.

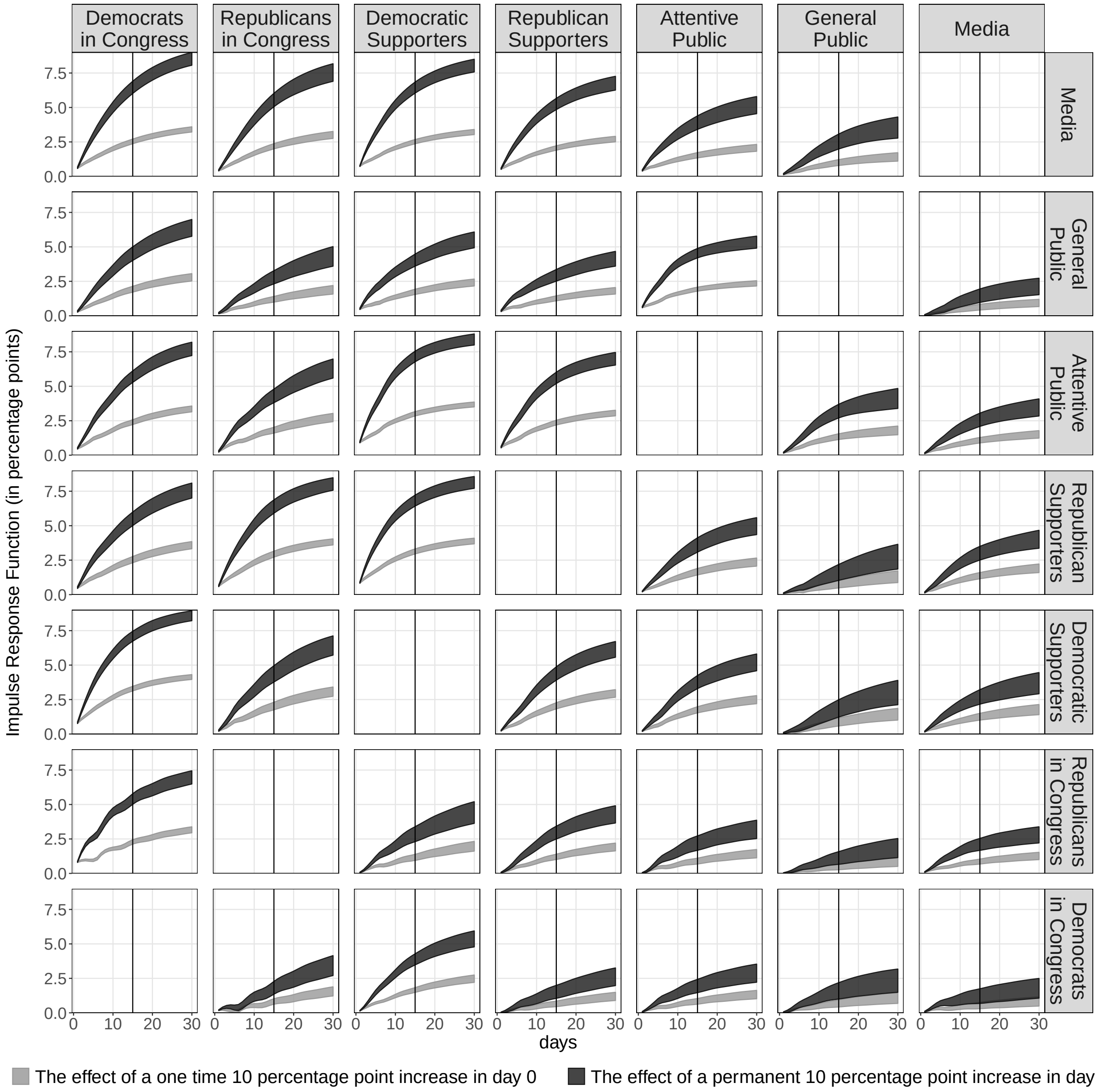

Note: The lines in the panels (with 95% confidence intervals) indicate how much more attention the groups in the panel titles pay to a given issue up to 60 days after the groups in the y-axis increased the attention to the same issue by 10 percentage points. The vertical line in each panel indicates the 15-day Cumulative IRFs reported in the paper (Figure 2).

APSR Submission Template

APSR Submission Template

E. ROBUSTNESS CHECKS

In this Appendix we evaluate the extent to which the results presented in the paper depend on a set of modeling choices. In particular, we evaluate the effects of: **a)** having in our sample members of Congress that were up for re-election in 2014 (all House representatives plus a third of the Senators) and Senators that were not (we compare our results when running the model with 2013- and 2014-only data), **b)** using broader rather narrow political issue categories (we compare our results to results based on mapping our issues to the *Comparative Policy Agendas* (CAP) major issue categories), **c)** using narrow topics from an unsupervised model instead of topics from a supervised approach that are based on an existing classification of narrow political issues (we compare the results in the paper to results based on mapping our issues to the CAP minor issue categories), **d)** fitting the LDA model only to tweets from members of Congress instead of the tweets from all the groups in the study (politicians, the public, and the media).

E.1. Exploring Election-Year Effects: Modeling 2013 and 2014 Data Separately

Theoretical accounts (Fiorina, 1973, *American Politics Quarterly*; Mayhew, 1974, *Congress: The electoral connection*) and empirical findings (Soroka and Wlezien 2009; Gilens 2012) indicate that politicians should be more responsiveness to the issue and policy preferences of the public in election years. In this Appendix we first check whether that is the case (its is: politicians are more likely to be responsive to the general public during election years), and then, we explore whether the main findings of the paper hold when looking at results from election *versus* non-election years (they do).

At the end of 2014, the second year of the 113th Congress (our period of analysis), there was a mid-term election and so all House representatives and a third of the Senators were up for re-election. In a study of policy (not issue) responsiveness in the United States, Gilens (2012) shows that public representatives respond to the policy preferences of the general public only in election years. We follow a similar strategy and use the same exact dataset used in the paper (time-series indicating the attention that Democrats in Congress, Republicans in Congress, Democratic Supporters, Republican Supporters, Attentive Publics, General Public, and the Media paid to 46 political issues) and compare results for the the main VAR model of the paper (Equation 1) when only fitting it to data from a non-election year (2013) *versus* fitting it to data from an election year (2014).

Figure A5 shows the two new model results. Overall, we do not see strong election-year effects. In the two most left panels we observe Democrats and Republicans in Congress to show similar degrees of responsiveness in 2013 and 2014: there is an overlap between most gray and black coefficients. For example, in both years Democrats in Congress were equally responsive to their party supporters. There is however one noticeable difference. In line with Gilens (2012) and Soroka and Wlezien (2009)'s findings on policy (not issue) preferences, we do observe that members of Congress are more likely to respond to the issue preferences of the general public during election years: the gray and black

FIGURE A5. Comparing VAR results and responsiveness dynamics from election (2014, darker coefficients) *versus* non-election years (2013, lighter coefficients).

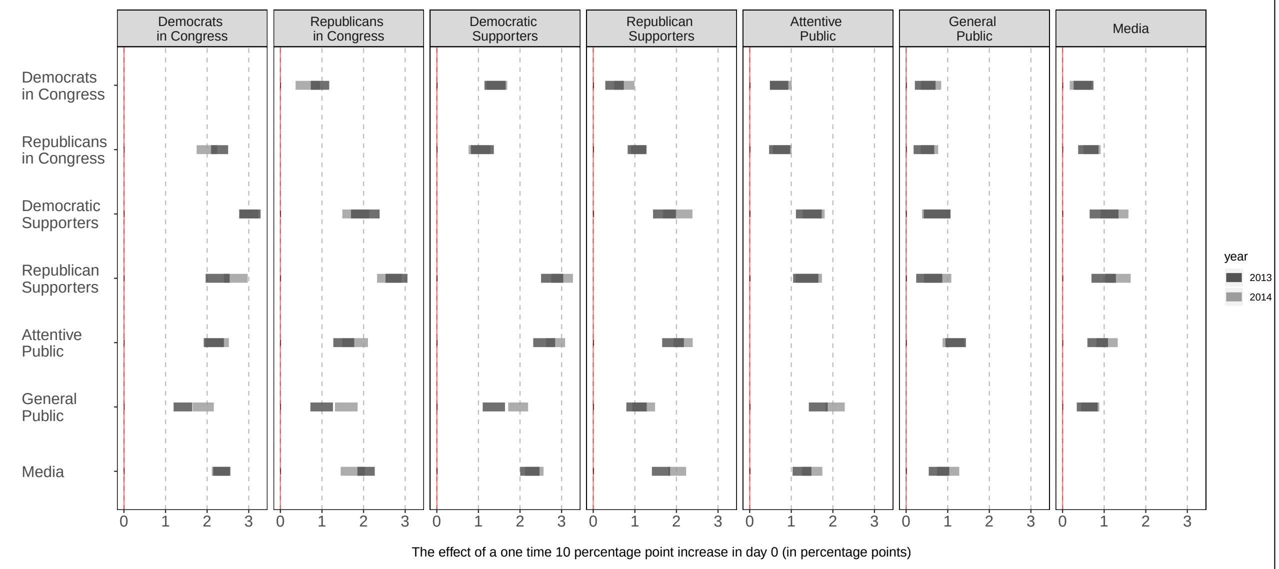

Note: The coefficients indicate (in percentage points) how much more attention the groups in the panel titles pay to a given issue 15 days after the groups in the y-axis increased the attention to the same issue by 10 percentage points.

coefficients for the General Public in the two most left panels do not overlap. The findings support the argument that when they are not facing an electoral contest, public representatives are mostly responsive to their immediate and mobilized constituents, such as their party base, attentive constituents and interest groups. Nevertheless, in order to increase their chances of reelection, they do pay a bit more attention to the issues preferences of the general public during election years. In Figure A5 we observe how in 2014 the ability of the general public to set the issue agenda of members of Congress was similar to the ability of attentive voters and the media, whereas in 2013 it was much lower than any other group of the public or the media.

Finally, the results in Figure 12 indicate that the main findings of the paper hold when fitting the model to data from an election-year only: we still observe some the public to have a stronger ability to set the issue preferences of members of Congress than the other way around; and we still see members of Congress to be mostly responsive to the issue preferences of their party supporters and attentive public, although we do observe the general public to have a bit more influence on the political agenda during election years.

E.2. Modeling Broader Political Issues

Topics from an unsupervised 100-topic model are of a narrow scope. For example, instead of a broad immigration topic, we discovered a topic on comprehensive immigration reform and a topic on President Obama's executive action on immigration. The advantage of focusing on narrower topic

APSR Submission Template APSR Submission Template APSR Submission Template APSR Submission Template APSR Submission Template APSR Submission Template APSR Submission Template APSR Submission Template

definitions is that we can study attention to specific important issues that dominated the public, media and political agenda for a relevant period of time, and that we can more easily study whether the public responded to a political change in attention by politicians or the other way around.

Focusing on narrow topics has a potential drawback. The goal of the study is to learn about the type of publics politicians are responsive to. If topics are too narrow, we run the risk of studying attention to party frames (how Democrats or Republicans talk about a given issue) instead of topics. This may influence our results in favor of the supporter model and in detriment of the attentive and Downsian arguments.

In the paper we addressed this potential problem by merging issues from the topic model that were closely related: 2 sub-issues about student debt, 2 about the Hobby Lobby Supreme Court decision, 2 on budget discussions, and 5 about the 2013 Government shutdown. In here we run a robustness check to evaluate the extent to which our results are a function of studying specific instead of broader issues.

FIGURE A6. A comparison between the VAR results in the paper and the VAR results of a model exploring attention to broader political issues (Comparative Policy Agendas issue classification).

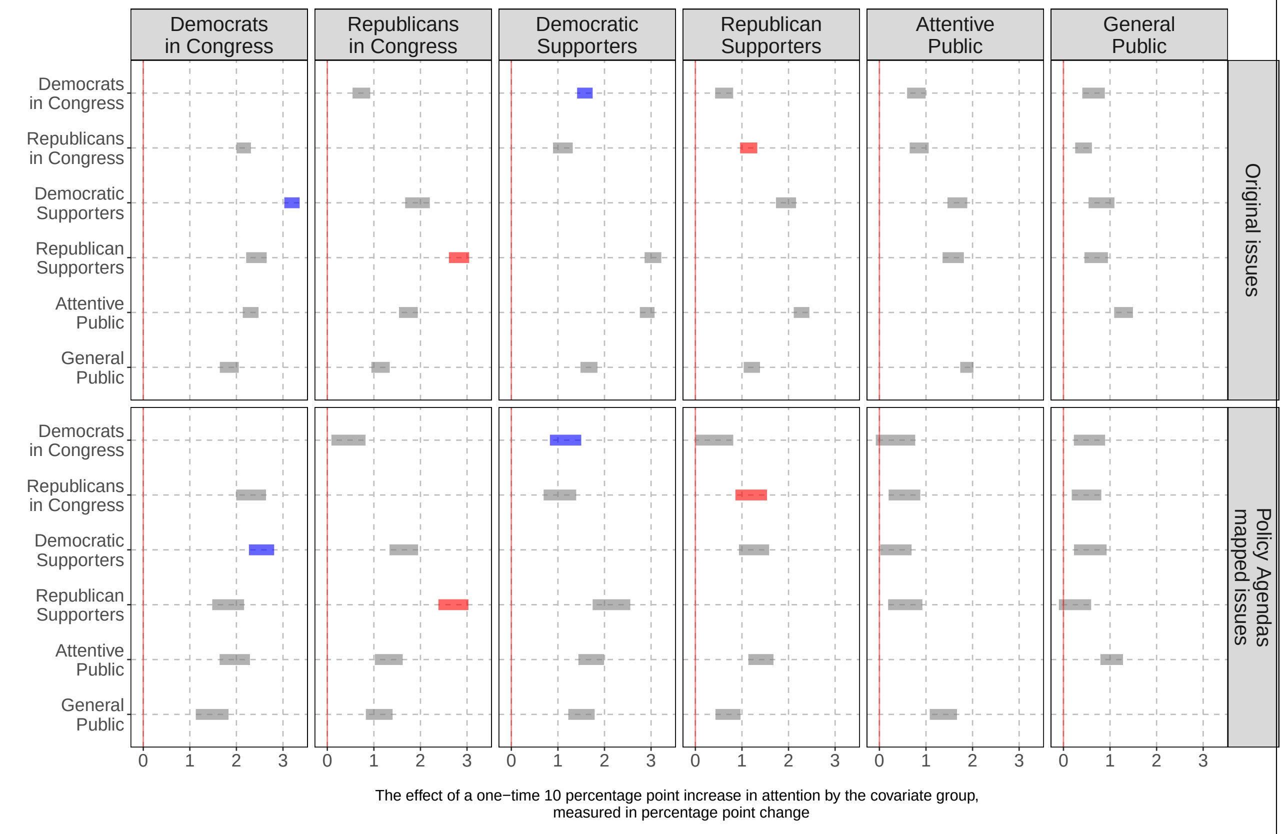

Note: The coefficients indicate (in percentage points) how much more attention the groups in the facet titles pay to a given issue 15 days after the groups in the y-axis increased the attention to the same issue by 10 percentage points.

First, we use the crosswalk Table A1 from the *Validation of Discovered Topics* Appendix to map

APSR Submission Template

each of our 46 political issues to a issue-classification based on much broader issues: the 19 major topic classification of the Comparative Agendas Project (CAP).[3] Then we re-estimate the same VAR model presented in the paper (Equation 1). In Figure A6 we compare the results we presented in the paper (six top panels) to the results of a VAR model studying attention to the Comparative Policy Agendas topics (six bottom panels).

Three main points stand out. First, when modeling attention to CAP major topics we still observe members of Congress to be first responsive to their party supporters (for Democrats: blue estimates in the top and bottom left panels. For Republicans: red estimates in the second from the left top and bottom panels), and then to attentive voters (attentive publics and supporters of the other party). Second, as we saw in Figure 3 in the paper, we still observe the ability of the public to influence the attention distribution of politicians to be higher than the *vice versa* effect: the blue estimates in the two left panels are of larger magnitude than the blue estimates in the third from the left panels, and the red estimates in the second from the left panels are of larger magnitude than the ones in the third from the right panels. Finally, in this new model results we still observe the general public to pay a residual role. They have little ability to set political agendas (bottom estimates in bottom and top left panels) and they do not positively respond to changes in attention by members of Congress.

Overall, the results of the new model show that the main findings presented in the paper hold when modeling attention to broader issues instead of more specific ones.

E.3. Modeling an Existing Classification of Narrow Political Issues

The Comparative Agendas Project has also developed a set of minor issue codes for each of the major issue categories, breaking the 19-issue classification into a 314-(minor)-issue categorization. Hence, instead of using an unsupervised method (LDA) to discover a set of narrow political topics, we could have used the CAP minor issue codes to manually label a set of tweets from our study and then train supervised machine learning classifiers capable of automatically classifying the rest of the tweets.

We decided not to take this path for two main reasons. First, despite the large number of minor topic codes, some are still of a broad nature. For example, there is only one minor *Immigration* topic but, as addressed in the previous subsection, we have discovered that members of Congress discussed more than one immigration-related issue during the 113th Congress. Moreover, training accurate classifiers capable of predicting all minor topic codes would have required to manually label an incredibly large number of tweets.

Nevertheless, here we follow the same procedure described in E.2 to assess whether we reach similar conclusions when we map our issues to the CAP minor issue codes and then re-run the analysis (see Table A1 for the CAP minor topic codes assigned to each of our topics). In Figure A7 we compare the original results in the paper (top six panels) to the results that originate from mapping our issues to the CAP minor issue codes (six bottom panels). The results are essentially the same. Politicians

[3] http://comparativeagendas.net/

APSR Submission Template APSR Submission Template APSR Submission Template APSR Submission Template APSR Submission Template APSR Submission Template APSR Submission Template APSR Submission Template APSR Submission Template

APSR Submission Template

FIGURE A7. A comparison between the VAR results in the paper and the VAR results of a model exploring attention to the minor issue codes of the Comparative Policy Agendas classification.

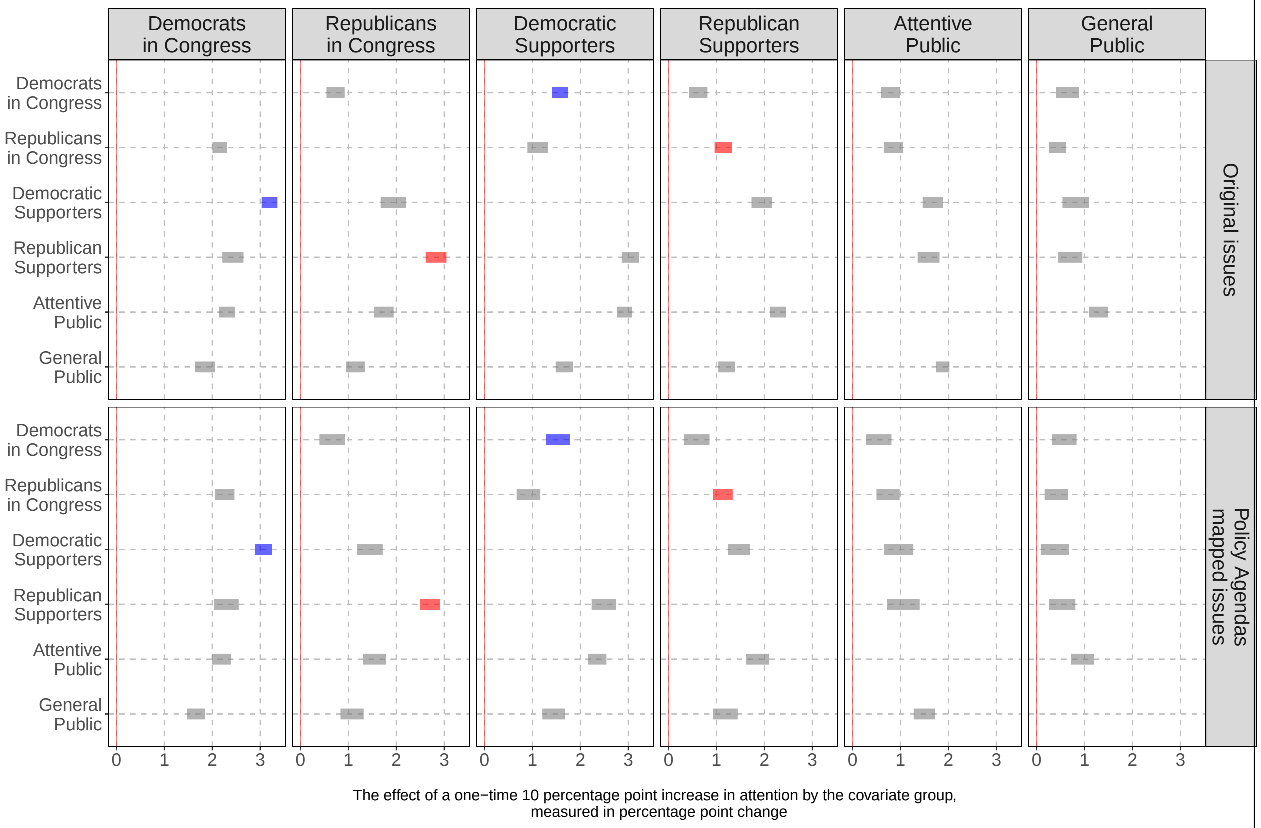

Note: The coefficients indicate (in percentage points) how much more attention the groups in the facet titles pay to a given issue 15 days after the groups in the y-axis increased the attention to the same issue by 10 percentage points.

have the ability to influence the attention that the public pays to different political issues, and *vice versa*. However, the ability of the public to influence the issue attention of politicians is slightly greater. Politicians are particularly responsive to changes in issue attention by their party supporters and attentive publics.

E.4. Modeling Topics Discovered in All Tweets

In the paper we select the set of political issues to study by fitting an LDA model to the tweets of members of Congress. We then assess the extent to which the attention that politicians, the public, and the media pay to the resulting issues can be explained by an increase or decrease in attention by the other groups; indicating the presence of issue-responsiveness dynamics.

The advantage of fitting the LDA model only to the tweets of members of Congress is that we are more likely to discover political (rather than non-political) topics: research clearly shows that the mass public pays little attention to politics (Carpini and Keeter 1996; Hibbing and Theiss-Morse 2002) and

FIGURE A8. Goodness of fit statistics for several LDA models (with different number of topics) fit to all tweets from politicians, the media, and the public.

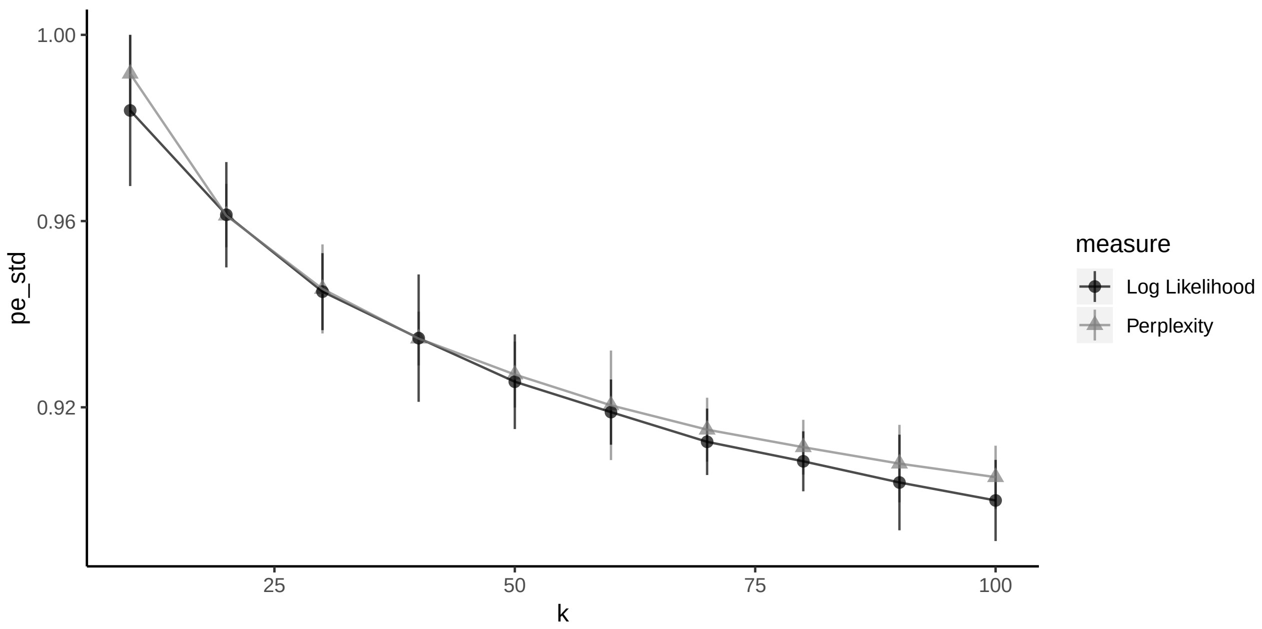

that when it does, it tends to focus on a small set of issues (Jones and Baumgartner 2004). Fitting an LDA model to all tweets from politicians, the media, and the mass public would hence lead to missing some political topics in detriment of non-political issues; which we clearly wanted to avoid.

A disadvantage of the approach used in the paper is that we could be potentially missing political topics discussed by the public but not by members of Congress. Although research indicates that this scenario is rare Carpini and Keeter (1996); Hibbing and Theiss-Morse (2002); Jones and Baumgartner (2004), in this section we examine whether we would have reached similar conclusions if we had initially fit an LDA model to the combined tweets of politicians, the media, and the public, instead of to the tweets of only politicians.

First, we fix the number of topics of the new LDA model by running several LDAs with a different number of topics k to all tweets of the study and examining the log likelihood and perplexity on holdout samples using 5-fold cross-validation. Similar to Figure A12 in the paper, Figure A8 shows these goodness of fit measures as the number of topics in the x-axis increases from 10 to 100. Similar to the original LDA model, we observe convergence for values of k as these get close to 100 and so we chose to fit another 100-topic LDA model to avoid over-fitting.

Table A4 illustrates the 100 topics discovered by this new LDA model. Few general traits stand out. First, as expected (and as indicated in the *Political* column of the table), we discover fewer political topics in this model than in the model used in the paper: 34 v. 53, respectively (27 v. 46 after merging the topics in each model that are very similar). Second, we observe that 20 of the 27 political topics in this new model are also present in the original topic model used in the paper. There are however 7 topics that were not discovered in the original LDA model, indicating that the mass public or the media

APSR Submission Template

FIGURE A9. A comparison between the VAR results in the paper (in black) and the VAR results based on a 100-topic LDA model fit to the combined tweets of politicians, the media, and the public (in gray).

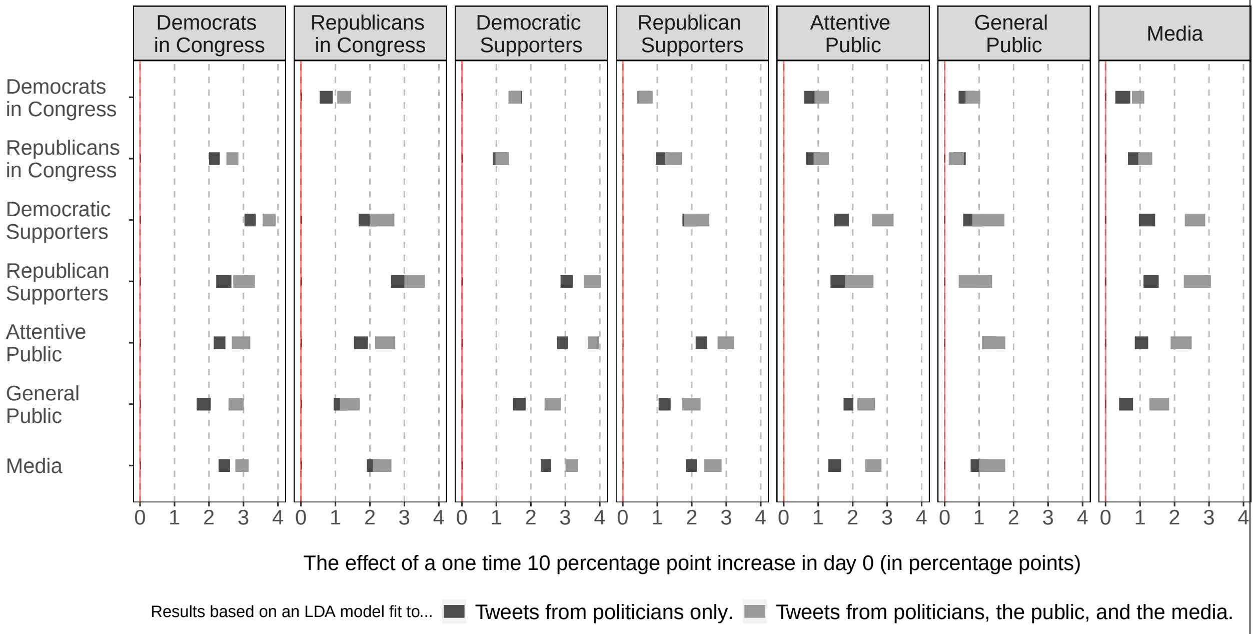

Note: The coefficients (with 95% confidence intervals) indicate (in percentage points) how much more attention the groups in the facet titles pay to a given issue 15 days after the groups in the y-axis increased the attention to the same issue by 10 percentage points.

paid a larger attention to them than politicians did: 2 topics related to the Black Lives Matter (BLM) movement (one about the incidents in Ferguson and another one about police brutality more generally), 3 foreign affairs topics (on Bowe Bergdahl, the Israel-Palestine conflict, and on defense and foreign policy generally), and two topics on national politics (one on Chris Christie and another one about local economies).

We fit the same VAR model (Equation 1) to the time series generated by these new 27 political topics, and present the results in Figure A9, where the original results are shown in black and the new results are shown in gray. The key inferences from the new estimates are generally consistent with the inferences from the original estimates: both Democratic and Republican members of Congress are primarily responsive to changes in attention by their party supporters and attentive voters, and their ability to influence changes in attention by these groups is lower than the *vice versa* effect.

There are some differences worth noticing when we compared to the original results. First, we observe in the new results that the ability of the public and the media to influence the agenda of members of Congress is slightly higher (the gray coefficients in the left two facets are higher than the black ones). Second, we observed that the ability of the media and the different groups of the public to influence the issue agenda of other groups of the public is also higher (the gray coefficients for party supporters, attentive public, general public, and the media are higher in the three middle facets). And

APSR Submission Template

finally, in the same way we observed the media to have a larger issue agenda setting role, we also observe the public to have a higher ability to set the issue agenda of the media (the gray coefficients for party supporters, attentive public, and general public are higher in the panel on the right). These two final points suggest that, by not fitting the original model to the tweets of the public and the media, in the original results we might have slightly underestimated the intermediate agenda setting role of the media. Nevertheless, the core findings of the study remain the same, and by focusing only on the topics discovered in the tweets of members of Congress, we were able to provide more detailed information about a larger number of political topics legislators discussed during the 113th Congress.

E.5. Rulling out a Media-Collider Bias

In our VAR models we control for the attention that media outlets pay to the issues under study in order to control for potential media effects and the alternative explanation that media outlets may be the actors leading changes in political and public issue attention. However, since we observe both politicians and the public to lead media attention, there is room for a potential collider bias, and so for the observed relationship between groups of the public and politicians to be a simple artifact of controlling for media effects. Here we rule out this possibility by fitting the main VAR model of the paper without controlling for media effects. In Figure A10 we compare the IRFs of two models, one in which we control for media effects (in blue), and another one in which we do not (in orange). We observe that the predicted relationships between the different groups of the public and politicians do not vary.

FIGURE A10. Comparing the results for the main VAR model of the paper when controlling, and not controlling, for media effects.

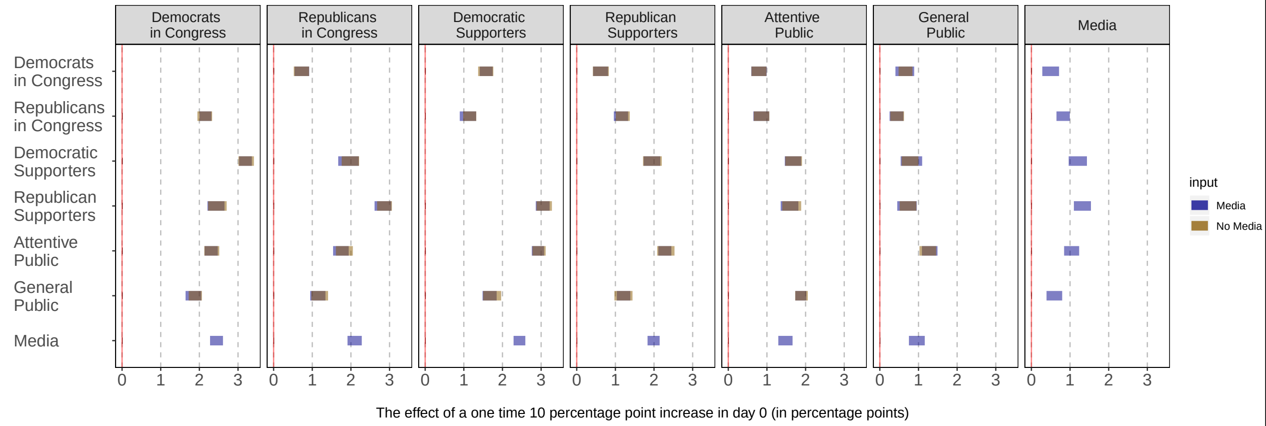

APSR Submission Template APSR Submission Template APSR Submission Template APSR Submission Template APSR Submission Template APSR Submission Template APSR Submission Template APSR Submission Template

TABLE A4. Description of the topics in the alternative 100-topic LDA model fit to all tweets from politicians, the media, and the public. The *Political* column indicates whether we considered to topic to be a political issue, and the *Match* column indicates whether the same (or a very similar) topic exists in the LDA model used in the paper

#	Most Predictive Features	Label	Political	Match
8	#obamacare, hearing, meeting, #tcot, passed, icymi, rep, obamacare, budget, staff	Obamacare	Yes	Yes
9	gop, tax, republicans, budget, party, voted, rep, votes, pay, republican	Government Budget	Yes	Yes
11	@sentedcruz, #makedclisten, cruz, obamacare, #defundobamacare, @senrandpaul, #standwithrand, ted, ted cruz, rand	Government Shutdown	Yes	Yes
17	nsa, father's, father's day, #nsa, snowden, june, immigration, happy father's, fathers, surveillance	NSA Surveillance Scandal	Yes	Yes
18	marriage, court, gay, samesex, gay marriage, supreme, equality, supreme court, samesex marriage, marriage equality	Marriage Equality	Yes	Yes
20	zimmerman, trayvon, black, martin, george, trayvon martin, george zimmerman, justice, verdict, white	Police Brutality - BLM	Yes	No
24	climate, isis, march, change, #peoplesclimate, scotland, nfl, climate change, sept, #indyref	Climate Change	Yes	Yes
30	city, business, million, free, post, county, high, latest, tomorrow, stay	Local Economy	Yes	No
31	christie, 2014, snow, cold, jan, unemployment, chris, january, winter, weather	Chris Christie	Yes	No
35	ukraine, march, putin, russia, #ukraine, russian, crimea, flight, patrick's, spring	Protests in Ukraine	Yes	Yes
38	israel, gaza, hamas, #gaza, border, israeli, #israel, children, war, killed	Israel-Palestine	Yes	No
40	#tcot, gun, god, #tgdn, control, guns, media, follow, 2013, gun control	Gun Violence	Yes	Yes
41	isis, 9/11, labor, #neverforget, #isis, joan, labor day, strategy, rivers, september	Islamic State	Yes	Yes
42	bergdahl, cantor, june, taliban, #bergdahl, eric, bowe, california, campaign, bring	Bowe Bergdahl	Yes	No
46	election, gop, voting, voters, republican, republicans, polls, win, voted, race	Election Day 2014	Yes	Yes
51	rep, #raisethewage, let's, #renewui, economy, reform, community, million, #aca, pay	Minimum Wage	Yes	Yes
54	syria, #syria, war, chemical, weapons, syrian, attack, labor, chemical weapons, kerry	Use of Military Force in Syria	Yes	Yes
59	pope, sequester, march, budget, cuts, white, #sequester, paul, francis, sequestration	2013 Budget Sequestration	Yes	Yes
60	irs, #irs, scandal, holder, benghazi, #benghazi, targeting, groups, tea, party	IRS Scandal	Yes	Yes
63	#ferguson, ferguson, police, black, brown, grand, wilson, jury, grand jury, darren	Freguson - BLM	Yes	No
65	gun, guns, gun control, control, violence, nra, sandy, 2013, gun violence, debt	Gun Violence	Yes	Yes

APSR Submission Template APSR Submission Template APSR Submission Template APSR Submission Template APSR Submission Template APSR Submission Template APSR Submission Template APSR Submission Template

67	border, #tcot, illegal, irs, emails, iraq, illegals, #bringbackourmarine, lost, #pjnet	Immigration	Yes	Yes
68	#benghazi, benghazi, mother's, mother's day, mothers, mom, happy mother's, hillary, moms, draft	Investigation of Attack on American Embassy in Benghazi	Yes	Yes
74	obamacare, navy, yard, #breakingbad, navy yard, shooting, #obamacare, gun, ios, @andi_sloan	Obamacare	Yes	Yes
77	shutdown, government, gop, #shutdown, obamacare, debt, republicans, shut, government shutdown, #gopshutdown	Government Shutdown	Yes	Yes
79	ebola, #ebola, cdc, patient, dallas, africa, texas, travel, october, hospital	Ebola	Yes	Yes
83	war, bush, party, john, deal, win, obama's, gop, attack, say	Defense and Foreign Policy	Yes	No
85	hobby, lobby, hobby lobby, #hobbylobby, court, supreme, #scotus, supreme court, control, decision	Hobby Lobby Supreme Court Decision	Yes	Yes
86	#tcot, isis, #pjnet, god, islamic, obama's, 2014, #isis, @jjauthor, muslim	Islamic State	Yes	Yes
87	immigration, obama's, executive, #immigration, action, #immigrationaction, amnesty, @barackobama, immigrants, keystone	Executive Action on Immigration	Yes	Yes
88	#ericgarner, police, #icantbreathe, #blacklivesmatter, torture, eric, garner, black, cia, eric garner	Police Brutality - BLM	Yes	No
92	obamacare, #obamacare, insurance, website, plans, health care, iran, sebelius, healthcare, health insurance	Obamacare	Yes	Yes
95	texas, #txlege, #standwithwendy, #sb5, rights, #scotus, wendy, court, @wendydavistexas, #doma	Abortion	Yes	Yes
98	#ferguson, police, ferguson, black, brown, #mikebrown, cops, michael, officer, michael brown	Freguson - BLM	Yes	No
14	#tcot, obamacare, god, 2014, follow, gun, #obamacare, #pjnet, government, life	Party Talk	No	No
15	#uniteblue, 2014, #p2, gop, #tcot, #renewui, black, republicans, htt ..., wage	Party Talk	No	No
25	#tcot, #pjnet, gruber, amnesty, obama's, obamacare, @foxnews, stupid, #gruber, black	Party Talk	No	No
50	gun, #p2, 2013, #uniteblue, gop, #getglue, stories today, social, gay, background	Party Talk	No	No
55	#tcot, 2014, #pjnet, gun, god, free, #teaparty, reid, harry, government	Party Talk	No	No
69	#uniteblue, #p2, gop, 2014, htt ..., #tcot, republicans, @dailykos, gun, htt	Party Talk	No	No
75	gop, #p2, 2013, #uniteblue, republicans, food, #tcot, party, media, #gop	Party Talk	No	No
81	#tcot, obamacare, god, @sentedcruz, obama's, #benghazi, follow, @breitbartnews, media, @foxnews	Party Talk	No	No
93	august, obamacare, nsa, weiner, aug, black, baby, egypt, royal, stories today	Party Talk	No	No
97	2014, #uniteblue, black, gop, htt ..., #p2, @dailykos, republicans, white, 2015	Party Talk	No	No

APSR Submission Template APSR Submission Template APSR Submission Template APSR Submission Template APSR Submission Template APSR Submission Template APSR Submission Template APSR Submission Template

1	memorial, memorial day, #yesallwomen, #memorialday, maya, angelou, maya angelou, men, veterans, remember	Memorial Day	No	Yes
2	#gameinsight, i've, #androidgames, #android, #androidgames #gameinsight, #gameinsight i've, #android #androidgames, collected, coins, gold	Entertainment	No	No
3	2014, photo, foto, follow, 2013, htt ..., haha, ang, @estucrudaverdad, snow	Entertainment	No	No
4	follow, #iphone, #iphonegames #gameinsight, #iphonegames, haha, #iphone #iphonegames, followers, love, #gameinsight, stats	Entertainment	No	No
5	halloween, #halloween, #worldseries, series, costume, world series, happy halloween, game, nov, candy	Halloween	No	No
6	think, can't, did, say, really, that's, man, got, you're, does	Boilerplate	No	No
7	super, bowl, valentine's, super bowl, valentine's day, valentines, #superbowl, valentines day, love, power	Super Bowl and Valentine's Day	No	1
10	follow, @camerondallas, love, bae, summer, #callmecam, birthday, #mtvhottest, 2014, mean	Entertainment	No	No
12	follow, love, bae, birthday, happy birthday, life, mean, 2014, game, boys	Entertainment	No	No
13	follow, haha, love, thirsty, lol, 2013, thirsty thirsty, followers, spring, snow	Entertainment	No	No
16	photo, posted, facebook, photo facebook, new photo, posted new, facebook posted, love, weekend, photos	Photos Posted on Facebook	No	Yes
19	bowl, super, super bowl, #superbowl, broncos, game, seahawks, peyton, #sb48, seattle	Super Bowl XLVIII	No	No
21	boston, marathon, suspect, bombing, police, boston marathon, #boston, #bostonmarathon, gun, explosion	Boston Marathon	No	Yes
22	spring, march, #marchmadness, bracket, win, ncaa, game, april, basketball, michigan	March Madness	No	No
23	#oscars, #goldenglobes, oscar, @theellenshow, oscars, photo, longer, arm, #oscars2014, best photo	Oscars	No	No
26	que, los, por, para, las, del, una, mas, como, esta	Spanish Twittershpere	No	No
27	snow, #sochi2014, olympics, usa, feb, sochi, winter, olympic, #wearethepeople, gold	2014 Winter Olympics	No	Yes
28	cup, world cup, #worldcup, usa, #usa, game, #worldcup2014, iraq, #usmnt, soccer	World Cup	No	Yes
29	follow, love, followers, birthday, stats, unfollowers, today stats, followed, mean, snow	Entertainment	No	No
32	change, love, tell, community, rights, climate, we're, public, education, action	Community	No	No
33	man, say, police, #edshow, white, photos, here's, breaking, death, photo	Breaking News	No	No
34	game, football, team, win, play, auburn, alabama, sec, college, fans	College Football	No	No
36	jeter, holder, october, derek, eric, oct, game, secret, derek jeter, hong	Baseball	No	No

37	police, cops, nypd, officers, #nypd, cuba, sony, cop, mayor, black	Unclear	No	No
39	game, football, nfl, win, team, season, play, cowboys, sunday, fans	Football	No	No
43	night, game, love, weekend, come, saturday, photo, win, birthday, fun	Entertainment	No	No
44	love, lol, got, can't, really, you're, shit, life, think, fuck	Entertainment	No	No
45	class, tomorrow, come, game, friday, thursday, birthday, win, excited, #tbt	Entertainment	No	No
47	sunday, church, win, god, #nerdland, #uppers, race, watching, #nascar, life	Religion	No	No
48	monday, weekend, #thewalkingdead, tomorrow, happy monday, sunday, dead, win, mondays, #rhoa	Entertainment	No	No
49	thanksgiving, happy thanksgiving, thankful, black, black friday, friday, #thanksgiving, turkey, holiday, #blackfriday	Thanksgiving	No	Yes
52	robin, williams, robin williams, rip, suicide, #robinwilliams, sad, iraq, #riprobinwilliams, rip robin	Robin Williams's Death	No	No
53	2013, follow, #getglue, haha, love, photo, que, lol, hahaha, yang	Entertainment	No	No
56	love, life, @youtube, free, lol, you're, music, favorite, things, think	Entertainment	No	No
57	mandela, nelson, nelson mandela, #peopleschoice, #bethanymotagiveaway, #bethanymotagiveaway #bethanymotagiveaway, holiday, snow, jensen, 2013	Nelson Mandel's Death	No	Yes
58	2014, photo, @tm2000back, @myriammontecruz, men's, htt ..., dan, shi, update, following	Entertainment	No	No
61	lebron, heat, game, spurs, nba, james, miami, summer, june, finals	Basketball	No	No
62	sterling, donald, #bringbackourgirls, donald sterling, clippers, game, nba, bundy, girls, racist	Basketball	No	No
64	added, added video, video @youtube, @youtube playlist, playlist, @youtube, hot, historic, stock, hot new	Entertainment	No	No
66	rice, ray, ray rice, nfl, apple, iphone, violence, goodell, domestic, @nfl	Entertainment	No	No
70	4th, veterans, july, 4th july, independence, #veteransday, happy 4th, veterans day, fireworks, freedom	4th July	No	No
71	phil, duck, dynasty, duck dynasty, robertson, #duckdynasty, phil robertson, 2013, @aetv, free	Entertainment	No	No
72	#ff, friday, weekend, #scandal, happy friday, #tgif, @scandalabc, it's friday, #followfriday, sunday	Follow Friday Tweets (#FF)	No	Yes
73	#voicesave, #givingtuesday, paul, december, walker, kat, @nbcthevoice, #voicesave kat, paul walker, holiday	Entertainment	No	No
76	#grammys, king, luther, martin luther, martin, luther king, mlk, #inaug2013, dream, march	Martin Luther King	No	No
78	april, easter, happy easter, spring, game, jesus, fools, season, thatcher, april fools	Eastern	No	No

APSR Submission Template APSR Submission Template APSR Submission Template APSR Submission Template APSR Submission Template APSR Submission Template APSR Submission Template

APSR Submission Template APSR Submission Template APSR Submission Template APSR Submission Template APSR Submission Template APSR Submission Template APSR Submission Template APSR Submission Template

80	follow, summer, love, 2014, birthday, bae, followers, retweet, happy birthday, mean	Entertainment	No	No
82	tornado, cdt, issued, nws, oklahoma, warning, severe, june, cdt nws, april	Severe Wheather in Oklahoma	No	No
84	sen, senator, mcconnell, bipartisan, hearing, floor, sexual, assault, murray, reform	General Vocabulary (Senate)	No	Yes
89	christmas, merry, merry christmas, holiday, santa, holidays, eve, gift, #christmas, christmas eve	Christmas Holidays	No	Yes
90	#vote5sos, #votefifthharmony, #vmas, challenge, ice, bucket, ice bucket, 5sos, #votedemilovato, bucket challenge	Entertainment	No	Yes
91	christmas, follow, love, birthday, bae, 2014, 2015, #mtvstars, life, happy birthday	Entertainment	No	No
94	#sotu, state union, union, @barackobama, speech, rubio, wage, president obama, sotu, address	State of the Union Adress	No	Yes
96	new year, happy new, 2014, 2013, new years, year’s, new year’s, eve, cliff, resolution	New Year’s Eve	No	No
99	summer, july, photo, beach, park, hot, weekend, camp, june, follow	Entertainment	No	No
100	2014, 2015, photo, christmas, holiday, @youtube, htt ..., win, snow, direction	Entertainment	No	No

F. ADDITIONAL DESCRIPTIVE STATISTICS

F.1. Members of Congress on Twitter

This Appendix offers additional details regarding the data collection process. Our list of Twitter accounts of Members of Congress was collected through the New York Times Congress API and then revised for errors. We included only active Twitter accounts, which we consider to be those that sent at least one tweet during our period of analysis, although as shown in Figure A11, most legislators sent between 200 and 2,000 tweets during this period.

FIGURE A11. Distribution of number of tweets sent by Members of Congress during period of study

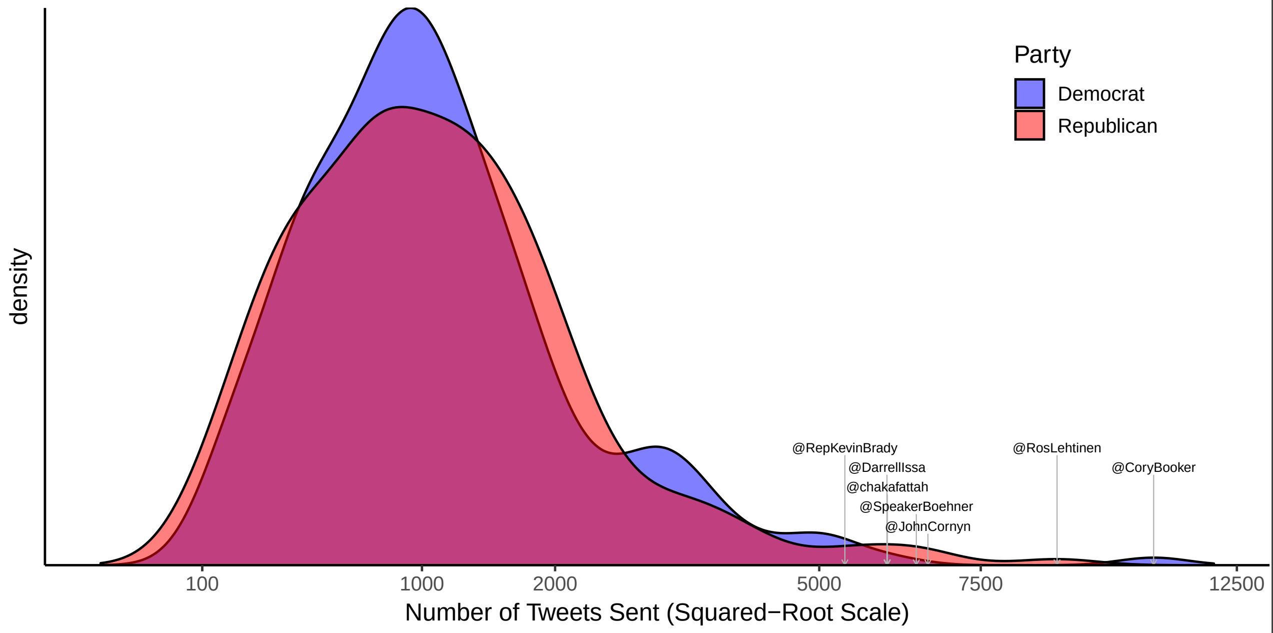

As noted in the main text, our data comprises all legislators that served during the 113th Congress. Multiple House representatives served in a few congressional districts: Jason T. Smith (MO-8), who won a special election in June 2013 after the previous incumbent resigned; David Jolly (FL-13), who substituted Bill Young; Catherine Clark (MA-5), who substituted Edward Markey after he was elected senator; Bradley Byrne (AL-1), who substituted Jo Bonner after he resigned; and Vance McAllister (LA-5), who substituted Rodney Alexander after his resignation. We also observe similar cases in the Senate: William Cowan, who substituted John Kerry as junior Senator from Massachusetts; Edward Markey, who substituted William Cowan after he declined to run in a special election; Jeffrey Chiesa, who substituted Frank Lautenberg as junior senator from New Jersey; and was in turn substituted substituted for Cory Booker; and John Walsh, who substituted Max Baucus after his appointment as U.S. Ambassador in China. We include in our dataset the tweets by legislators while they were in office.

APSR Submission Template

APSR Submission Template APSR Submission Template APSR Submission Template APSR Submission Template APSR Submission Template APSR Submission Template APSR Submission Template APSR Submission Template APSR Submission Template

F.2. Party Supporters on Twitter

Two of our citizen samples correspond to party supporters, which we identified as those that follow three or more members of Congress of one party and no legislators of the opposite party. In order to validate that this operationalization properly captures the notion of party supporters, we used data collected as part of a previous study (Barberá et al. 2015), where we matched geolocated Twitter accounts with voter registration records in five states (Arkansas, California, Florida, Ohio, and Pennsylvania) that make them publicly available for academic research purposes. From each of these datasets, we extracted party affiliation (Democratic or Republican party), turnout in the 2012 presidential election, and turnout in the 2010 congressional election; as well as the number of Members of Congress from each party that the voters follow on Twitter as of July 2018. Even if the data is more recent compared to our period of study, we believe it can provide useful evidence regarding the validity of our measurement strategy.

We find that our choice to identify party supporters as those who follow 3 or more members of Congress from one party and 0 from the opposite party is adequate. First, this threshold is able to classify party affiliation with approximately 90% accuracy: 87% (92%) of Twitter accounts in our sample who meet our criteria to be classified as a Republican (Democratic) supporter is affiliated with that party according to the voter files. Second, a large proportion (61%) of those who we identified as supporters turned out to vote in both elections (2010 and 2012). In contrast, turnout among voters affiliated with a party in our dataset was 51%. Finally, although this metric does not capture all party supporters, we find that 18% of voters who are affiliated with a party and voted in both 2010 and 2012 meet this definition.

We also considered alternative thresholds. If we increase the minimum number of accounts to 5, we see a minimal increase in the accuracy in predicting party affiliation (89% for Republicans; 93% for Democrats) and turnout among this group (63%), but the coverage of frequent voters by this metric drops by half (to 9%). If we lower the threshold to only one Member of Congress of a party and none of the other, we unsurprisingly see that around 55% of frequent voters meet this definition, but the metric now has high error rates when predicting party affiliation (with accuracy going down to 73% for Republicans and 86% for Democrats), and turnout is very similar to the entire population of voters who are affiliated with a party (54%). For these reasons, we think that our operationalization of party supporter is valid.

G. TOPIC MODELING OF TWEETS BY LEGISLATORS AND CITIZENS

G.1. Overview of Latent Dirichlet Allocation Model

Latent Dirichlet Allocation (LDA) treats each document as a random mixture over latent topics, and each topic as a probability distribution over tokens. Each document w in the corpus is the result of the following generative model (Blei et al. 2003, p.96):

1. The topic distribution for document w is determined by: $\theta \sim \text{Dirichlet}(\alpha)$
2. The token distribution for topic k is determined by: $\beta \sim \text{Dirichlet}(\delta)$
3. For each of the tokens in document w
   (a) Choose a topic $z_n \sim \text{Multinomial}(\theta)$
   (b) Choose a token w_n from $p(w_n|z_n, \beta)$, a multinomial probability conditioned on z_n.

The LDA model considers each document as a sequence of N tokens (which in our case are n-grams, or combinations of one and two words), denoted by $\mathbf{w} = (w_1, w_2, \ldots, w_N)$, extracted from a vector of length V containing all possible tokens in the corpus.

This model requires us to fix K, the number of possible topics. There are two main parameters of interest: β, a matrix of dimensions $K \times V$ indicating the distribution of tokens over topics; and θ, a matrix of dimensions $K \times N$ indicating the distribution of topics over documents.

In our application, we fit the model with a collapsed Gibbs sampler (Griffiths and Steyvers, 2004, *PNAS*), implemented in R (Grün and Hornik, 2011, *Journal of Statistical Software*). We ran a single chain for 1,000 iterations. We apply the usual pre-processing text techniques (converting all words to lowercase and removing stopwords, all words shorter than 3 characters, and all n-grams that appear in less than 10 documents, but keeping hashtags and user handles), and then select as features the N=75,000 most frequent unigrams and bigrams.

G.2. Choosing the Number of Topics of the LDA Model

To fix the number of topics, we ran our model multiple times with different values of the number of topics (K), using 10-fold cross-validation and computing the log likelihood and estimated perplexity on the holdout sample (two common goodness of fit measures for LDA models, see Chang et al, 2009, NIPS – where smaller values indicate a better model fit). Figure A12 reports these two measures of model fit when estimating the model with different numbers of topics, from 10 to 130. We find that K=100 fits the data best. A higher value of K would minimize the loglikelihood and the perplexity measures, but we choose a conservative K in order to avoid overfitting (Hastie, Tibshirani, and Friedman, 2009, *The Elements of Statistical Learning*).

APSR Submission Template APSR Submission Template APSR Submission Template APSR Submission Template APSR Submission Template APSR Submission Template APSR Submission Template APSR Submission Template APSR Submission Template

FIGURE A12. LDA model fit with different number of topics

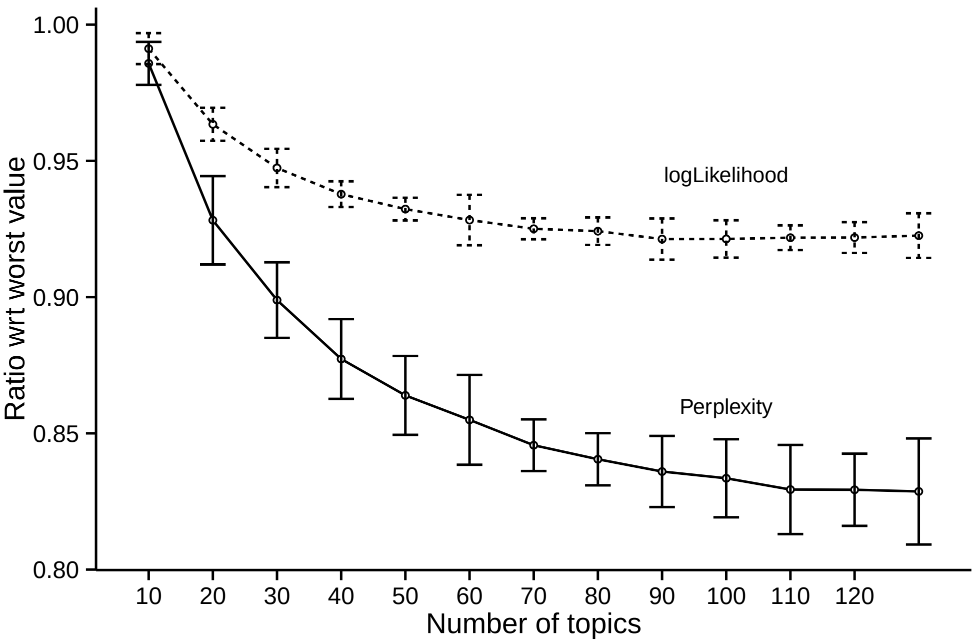

Note: This figure shows the cross-validated log likelihood and estimated perplexity after running our topic model with different numbers of topics. We find that 100 topics yields the best performance.

APSR Submission Template APSR Submission Template APSR Submission Template APSR Submission Template APSR Submission Template APSR Submission Template APSR Submission Template APSR Submission Template

G.3. Validation of Discovered Topics

In this Appendix we demonstrate that the topics that are discovered by the Latent Dirichlet Allocation model are valid representations of the political issues that legislators and citizens discussed during the 113th Congress. Following Quinn et al. (2010), we discuss how our results meet different notions of validity. First, we analyze the top scoring words for each topic to demonstrate that the topics that emerge from the model have a coherent meaning (semantic validity). Then, we examine whether topic usage corresponds correctly to external events (predictive validity). We will focus on whether topic usage is coherent with party identification for both legislators and citizens, and on whether spikes in their probability distribution can be matched to relevant political events.

To facilitate this validation exercise we have prepared an online appendix (or *dashboard*) where we offer a visualization of each of the topics that results from our analysis. The dashboard is available in the following URL: http://www.pablobarbera.com/congress-lda. A screenshot of one the topics is shown in Figure A13. We provide five different elements to interpret the issue that is associated with each topic: a plot indicating topic use by each of the groups we consider, the total estimated proportion of tweets from each group that belong to this topic, a graph with the top 15 n-grams most associated with that topic, the list of the five members of Congress who most often used this topic, and a sample of tweets by politicians and media outlets with a high probability to belong to this topic.[4]

As we show in Figure A13, it is easy to identify that this particular topic refers to debates about the minimum wage. From the time series plot, we learn that it started to be mentioned by Democratic legislators after January of 2014, when Barack Obama made this issue a central part of his State of the Union address, consistently with the notion of predictive validity. Democratic legislators and Democratic supporters are around 5 times more likely to discuss this topic than Republicans. The most common n-grams (#raisethewage, minimum wage, it's time, $10.10, workers, etc.), as well as the sample of tweets, are also related to this issue, which demonstrates the semantic validity of this topic.

Although not all topics have such a straightforward interpretation, in general we find that most topics that emerge from the analysis can be easily labeled. However, not all of them are political in nature: for example, we find topics about anniversaries and celebrations (Valentine's Day, Flag Day, Constitution Day, Thanksgiving, etc.). Since we are not interested in these topics, in our analysis we will only include political issues: we identified 53 of them (see Footnote 14). After reviewing their content, we noticed that some topics that referred to a single issue were classified as different topics because distinct words were being used by different groups when talking about the same issue. For example, we found separate separate topics for Republican and Democratic members of Congress discussing the 2013 Government Shutdown. This may influence our results by overestimating how often parties in Congress respond to their supporters. To avoid this potential source of bias, we decided to merge some topics and focus our analysis on 46 political issues. Table 2 displays the list of all these

[4]Note that although our topic model is fit using aggregated tweets, here the tweets were selected after computing the posterior probabilities at the tweet level.

topics we have classified as political issues.

We also compare the topics that emerge from the analysis to the list of key votes in Congress according to the Congressional Quarterly Almanac (see Table A5). This yearly publication selects a series of key votes in the House and Senate that are considered the "major issues of the year".[5] We find that only 16 (28%) out of 57 key votes in 2013 and 2014 cannot be matched to topics; and those that are not matched correspond to votes on relatively less important or less divisive issues, such as confirmations of presidential appointees, foreign policy decisions, and decisions on Senate rules. We also find that of the 46 political issues we identified in Table 2, 23 do not appear in the list of key votes, but in all cases because they're related to political action by other institutions (the Supreme Court or the President), or to external events, such as wars or attacks.

G.4. Attention to political issues by legislators and citizens

This Appendix complements the results shown in Figure 1, in which we can observe that the groups of the public do not pay an equal amount of attention to politics. These differences can be best appreciated in Table A6 below, where we show the average daily attention that each group dedicated to political topics during the 113th Congress. Members of Congress dedicated about 30% of their Twitter communications to discuss particular political issues. Party supporters also dedicated a substantive amount of their overall attention to discussing them: about 20%. Nevertheless, we observe the Attentive public, and particularly the General Public, to dedicate a much smaller fraction of their communications to discuss these political issues: 9% and 5% respectively.

Figure 1 (and Table A6) also highlights that mass media potentially played a key issue agenda setting role, as media outlets dedicated a large amount of attention to all the political topics that emerged during the 113th Congress. Moreover, we observe that, compared to the issue attention distribution of members of Congress, mass media distributed their attention more equally across topics. This is not surprising given that we included both liberal and conservative leaning outlets into our sample. Nevertheless, it is important to notice that, similar to mass media, party supporters also distributed their agenda more equally across topics, signaling a potential stronger relationship between their issue agendas.

[5]As defined in the publication, each vote is judged based on the extent to which it represents: 1) a major controversy, 2) a matter of presidential or political power, and 3) a potentially great impact on the nation and the lives of Americans.

APSR Submission Template

FIGURE A13. Visualization of Topics with Online Dashboard

Topic Usage Over Time:

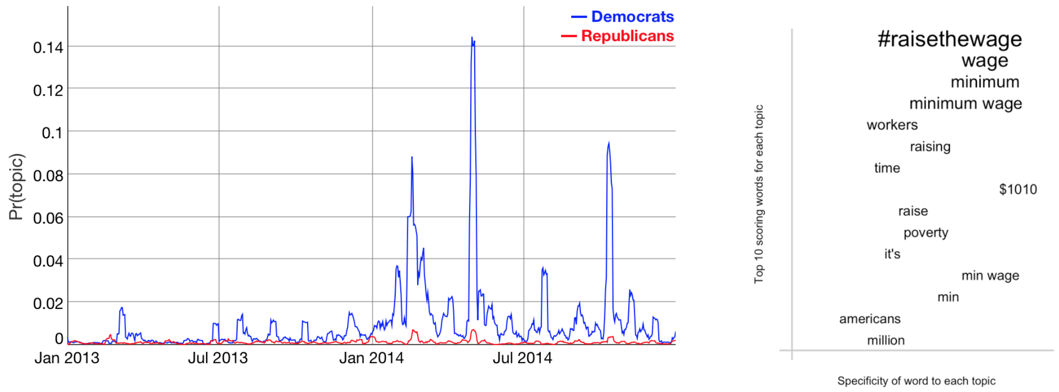

Display: ☑ Democrats ☑ Republicans ☐ Media ☐ Public ☐ Dem. Supporters ☐ Rep. Supporters ☐ Random Sample • **Smoothing period:** 7 days

Topic usage by elites: 0.59% all, 0.75% Senate Democrats, 0.20% Senate Republicans, 1.32% House Democrats, 0.09% House Republicans.
Top Members of Congress: @janschakowsky (D-IL), @keithellison (D-MN), @JuliaBrownley26 (D-CA), @SenatorHarkin (D-IA), @askgeorge (D-CA)
Topic usage by media and public: 0.52% Media, 0.07% informed public, 0.01% random users, 0.55% Democratic supporters, 0.14% Republican supporters.

Sample of representative tweets by Members of Congress:

Ed Markey @SenMarkey — Follow

Close to 2/3 of min wage workers are women. #RaiseTheWage will help nearly 15 million women & thousands in #MA.
4:35 PM - 24 Jul 2014
5 3

Nancy Pelosi @NancyPelosi — Follow

In marking 75 years of Fair Labor Standards Act, Congress must raise minimum wage so all workers get a fair day's pay for a fair day's work.
11:00 PM - 25 Jun 2013
111 34

Tom Harkin @SenatorHarkin — Follow

Today is the 75th anniversary of the minimum wage – it's time to #raisethewage again to help millions of Americans earn a living.
3:09 PM - 25 Jun 2013
31 5

Rosa DeLauro @rosadelauro — Follow

We need to respect workers and pass Fair Minimum Wage Act. Long past time to raise minimum wage to $10.10 #RaiseTheWage
7:11 PM - 25 Jun 2013
4 3

Sample of representative media tweets:

NBC News @NBCNews — Follow

Seattle's $15/hr minimum wage increase will be more than double the federal minimum wage of $7.25/hr. nbcnews.to/1wX5NGU
1:00 AM - 3 Jun 2014
151 84

Mother Jones @MotherJones — Follow

The GOP just a filibustered a bill to raise the minimum wage to $10.10.
Reminder: The minimum wage is pretty damn low bit.ly/1bbqLqq
2:00 AM - 1 May 2014
82 23

ThinkProgress @thinkprogress — Follow

How to raise nearly 5 million people out of poverty? Just raise the minimum wage to $10.10 thkpr.gs/1g5A90u
9:00 PM - 5 Jan 2014
60 25

Guardian US @GuardianUS — Follow

Restaurant workers are 3 times more likely to live below the poverty line than other Americans. Time for a raise trib.al/7wiGrvB
11:00 PM - 13 Feb 2014

APSR Submission Template

TABLE A5. Correspondence between key votes in Congress and our discovered political issues

2013 Key votes	Topics?	2014 Key votes	Topics?
H23 Superstorm Sandy Disaster Aid	**No**	H21 Omnibus Appropriations for 2014	103
H30 Debt limit	103	H30 Abortion Funding	16
H55 Violence Against Women Act	96	H31 Farm and Nutrition Programs	81
H89 Fiscal 2013 Appropriations	104	H61 Debt Limit	103
H125 Air Control Furloughs	64	H106 Climate Change Rules	50
H208 Immigration Enforcement	41	H156 Health Law Employer Mandate	63
H251 Abortion	16	H248 Medical Marijuana	**No**
H286 Farm and Nutrition Programs	81	H322 A-10 Airplanes	**No**
H325 Yucca Nuclear-Waste Storage	**No**	H327 Electronic Surveillance	20
H412 Electronic Surveillance	20	H452 Iraq Policy	**No**
H427 Iran Sanctions	**No**	H463 Endangered Species	**No**
H550 Government Shutdown	104	H507 Arming Syrian Rebels	33
H587 Health Insurance Implementation	63, 89	H519 Keystone XL Pipeline	39
H640 Budget Agreement	49	H550 Immigration Deportations	43
S24 Chuck Hagel Confirmation	**No**	H562 Tax Deductions for Charities	**No**
S92 Fiscal 2014 Budget Resolution	104	H563 Omnibus Appropriations for 2015	103
S97 Firearms Background Checks	15	S1 Janet Yellen Confirmation	**No**
S145 Farm and Nutrition Programs	81	S13 Omnibus Appropriations for Fiscal 2014	59
S168 Immigration Overhaul	67	S21 Farm and Nutrition Programs	81
S185 Student Loan Interest Rates	101	S33 Debt Limit	59
S199 Transportation-Hud Appropriations	**No**	S48 Debo Adegbile	**No**
S219 Government Shutdown	104	S59 Military Prosecutions	75
S232 Employee Nondiscrimination	28	S117 Minimum Wage	53
S242 Senate Filibuster Rules	**No**	S252 Child Migrants	**No**
S245 Defense Authorization	75	S262 Equal Pay for Women	9
S281 Budget Agreement	104	S280 Keystone XL Pipeline	39
		S282 Electronic Surveillance	20
		S354 Omnibus Appropriations for 2015	**No**
		S356 Surgeon General Nomination	**No**

Note: This table shows the topics in our model (second column) that corresponds to key votes in Congress (first column), as selected by the Congressional Quarterly Almanac. **No** indicates that a matching topic could not be identified.

TABLE A6. Percentage of the expressed issue agenda of different groups that was devoted to 46 political issues during the 113th Congress.

Group	Average Daily Attention to Political Topics
Democrats in Congress	27.28%
Republicans in Congress	27.08%
Democratic Supporters	19.26%
Republican Supporters	21.47%
Attentive Public	8.95%
General Public	5.33%
Media	32.14%

Note: The percentages represent the average of the sum of daily posterior probabilities-percentages assigned to political topics.

APSR Submission Template APSR Submission Template APSR Submission Template APSR Submission Template APSR Submission Template APSR Submission Template APSR Submission Template APSR Submission Template

REFERENCES

Barberá, Pablo , John T Jost, Jonathan Nagler, Joshua A Tucker, and Richard Bonneau (2015). Tweeting from left to right: Is online political communication more than an echo chamber? *Psychological science 26*(10), 1531–1542.

Blei, David M , Andrew Y Ng, and Michael I Jordan (2003). Latent dirichlet allocation. *the Journal of machine Learning research 3*, 993–1022.

Carpini, Michael X Delli and Scott Keeter (1996). *What Americans know about politics and why it matters*. Yale University Press.

Gilens, Martin (2012). *Affluence and Influence: Economic Inequality and Political Power in America*. Princeton, New Jersey: Princeton University Press.

Hibbing, John R. and Elizabeth Theiss-Morse (2002). *Stealth Democracy: Americans' Beliefs About How Governmnet Should Work*. Cambridge, UK: Cambridge University Press.

Jones, Bryan D. and Frank R. Baumgartner (2004). Representation and agenda setting. *Policy Studies Journal 32*(1), 1–24.

Quinn, Kevin M. , Burt L. Monroe, Michael Colaresi, Michael H. Crespin, and Dragomir R. Radev (2010). How to analyze political attention with minimal assumptions and costs. *American Journal of Political Science 54*(1), 209–228.

Soroka, Stuart N. and Christopher Wlezien (2009). *Degrees of Democracy: Politics, Public Opinion, and Policy*. Cambridge University Press.

APSR Submission Template APSR Submission Template APSR Submission Template APSR Submission Template APSR Submission Template APSR Submission Template APSR Submission Template
